# Probing NO_2_ Reactivity on Coinage Metal Surfaces through Liquid Crystal Orientational Responses

**DOI:** 10.1021/acs.jpcc.6c02631

**Published:** 2026-06-22

**Authors:** Evangelos Smith, Huaizhe Yu, Hanyu Zhang, Trenton J. Wolter, Alvaro Posada-Borbón, Robert J. Twieg, Nicholas L. Abbott, Manos Mavrikakis

**Affiliations:** † Department of Chemical and Biological Engineering, 5228 University of Wisconsin−Madison, Madison, Wisconsin 53706, United States; ‡ Department of Chemistry and Biochemistry, 4229 Kent State University, 1175 Risman Drive, Kent, Ohio 44242, United States; § Robert Frederick Smith School of Chemical and Biomolecular Engineering, 5922 Cornell University, 1 Ho Plaza, Ithaca, New York 14853, United States

## Abstract

Understanding the reactivity of nitrogen oxides (NO_*x*_) at interfaces remains a challenge for environmental monitoring and air quality control. Here, we show that 4-cyano-4'-pentylbiphenyl (5CB) liquid crystals (LCs) supported on coinage metal Au, Ag, and Cu surfaces exhibit NO_2_-induced orientational changes that provide an optical probe of NO_2_ adsorption and interfacial interactions on metal surfaces at parts-per-million (ppm) concentrations. Films of Au were prepared by electron-beam deposition and subsequently coated with submonolayer to multilayer films of Ag or Cu via electrochemical deposition. X-ray photoelectron spectroscopy reveals that these Ag and Cu films oxidize under ambient conditions to form oxygen-decorated Ag and Cu_2_O surface oxides, respectively. By combining polarized light microscopy, polarization-modulation infrared reflection–absorption spectroscopy (PM-IRRAS), and density functional theory (DFT) calculations, we show that 5CB adopts a planar orientation on Au and Cu overlayers on Au (Cu/Au) surfaces, but a perpendicular alignment on Ag overlayers on Au (Ag/Au). Upon exposure to 10 ppm of NO_2_ balanced in N_2_ at ambient pressure and temperature, 5CB undergoes substrate-dependent orientational transitions. On Au, a reversible planar-to-perpendicular transition is observed, which DFT calculations attribute to electrostatic stabilization of the perpendicular binding mode of 5CB by adsorbed NO_2_. On Cu/Au, the same transition in orientation of the LC occurs, but it is irreversible and correlates with the oxidation of Cu_2_O to CuO by NO_2_. Over ambient-exposed Ag/Au films, however, 5CB is unresponsive to NO_2_, retaining a perpendicular alignment before and after exposure. DFT calculations indicate that the presence of the LC modifies surface reaction thermodynamics on Ag/Au, shifting the preferred NO_*x*_ species formed from N_2_O_4_ in the absence of LCs to NO_2_ in their presence, and that NO_2_ adsorption does not alter the preferred orientation of 5CB. Taken together, these results demonstrate how substrate-dependent surface composition and oxidation state govern NO_2_ adsorption and speciation on coinage metal surfaces with and without LC overlayers.

## Graphical abstract

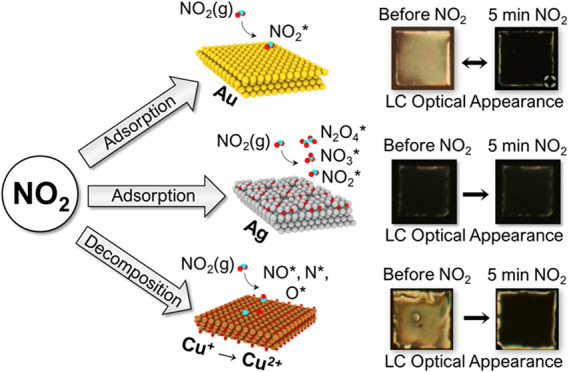

## 1 Introduction

Understanding reactions occurring on solid surfaces is fundamental to a wide range of environmental chemical processes (e.g., transformations of nitrogen oxides) as well as the development of new technologies for monitoring such processes (e.g., environmental sensors). Liquid crystalline (LC) films that respond to surface chemical reactions have emerged as a versatile platform for this purpose, capable of detecting trace analyte concentrations through orientational transitions induced by analyte interactions at solid-LC interfaces.^1–8^ These systems transduce atomic-scale events at surfaces into optically detectable changes in LC alignment. For example, strong-binding gas-phase organophosphorus compounds can displace nitrile-functionalized mesogens from coordination interactions with metal cations at salt-functionalized surfaces, shifting the orientations at which the LCs are anchored.^2,3,7^ Alternatively, oxidative gases, such as Cl_2_ and O_3_, can change the oxidation state and surface structure of metal substrates, triggering changes in the interactions and orientations of LCs at these surfaces.^1^ Building from these mechanisms, we have demonstrated that late transition metals and their alloys, including Pd–Au bimetallics, can drive chemically specific changes in LC alignment via dissociative adsorption of reactive species such as O_2_, Cl_2_, and H_2_.^4–6,9^

While prior studies have focused on Pd-based alloys, metal salts, and oxides for the design of interfaces to LCs that confer chemical responsiveness on the LCs, the group IB coinage metals, including Cu, Ag, and Au, remain largely unexplored in this context. Group IB coinage metals have been employed as substrates in sensing architectures and as electrical conductors,^10^ and they present a compelling opportunity to uncover new principles linking interfacial chemistry to macroscopic material responses. Moreover, coinage metals and their corresponding oxides exhibit diverse surface chemistries that drive industrially relevant processes. For instance, Ag is the catalyst of choice for the direct epoxidation of ethylene to ethylene oxide.^11^ Cu and its oxides serve as active components during methanol synthesis from syngas (CO/H_2_) and the water–gas shift (WGS) reaction.^12^ Although long considered catalytically inert, Au has more recently been shown to facilitate reactions such as CO oxidation.^13^

Beyond these catalytic roles, the surface chemistry of nitrogen dioxide (NO_2_) on coinage metals has also been extensively studied due to its environmental significance. NO_2_ is a toxic air pollutant produced primarily through fossil fuel combustion, contributing to respiratory disease and acid rain formation.^14^ On coinage metal surfaces, NO_2_ can undergo a wide range of surface-mediated processes: (1) reversible adsorption/desorption on Au,^15–17^ (2) partial dissociation on Ag and Cu,^18–20^ and (3) reaction with surface oxides to form nitrogen trioxide (NO_3_) or dinitrogen tetroxide (N_2_O_4_) on Ag.^21–23^ By coupling this rich surface chemistry with the orientational sensitivity of LCs to changes in interfacial interactions, we hypothesized that it would be possible to design optical sensors and soft actuators that provide a diversity of responses to NO_2_. Furthermore, we hypothesized that LC-based systems would generate new insights into the molecular-scale dynamics and reaction pathways of NO_2_ on Au, Ag, and Cu surfaces, potentially advancing both fundamental surface science and principles for environmental sensing.

In this study, we combine polarized light microscopy experiments, X-ray photoelectron spectroscopy (XPS) measurements, and density functional theory (DFT) calculations to elucidate the mechanisms underlying the orientational responses of 4-cyano-4'-pentylbiphenyl (5CB) LCs to NO_2_ when supported on as-deposited films of Au, Ag, and Cu. We observe that 5CB undergoes an optically detectable anchoring transition from planar to perpendicular (homeotropic) alignment upon exposure to NO_2_ when the LC is supported on ambient-exposed Au and Cu films. In contrast, 5CB remains unresponsive when supported on ambient-exposed Ag films, maintaining perpendicular alignment before and after NO_2_ exposure. DFT calculations reveal that favorable electrostatic interactions between NO_2_ and 5CB stabilize the perpendicular alignment of 5CB on Au. On Cu, the observed anchoring transition is attributed to oxidation of the Cu surface from Cu^+^ to Cu^2+^ by NO_2_. Finally, our mechanistic analysis shows that low equilibrium NO_*x*_ surface coverages on Ag films are insufficient to alter the perpendicular alignment of 5CB upon exposure to NO_2_. However, DFT predicts that the presence of the LC modifies surface reaction thermodynamics on Ag films to change the relative abundance of NO_2_ and N_2_O_4_ species. Overall, these results establish a direct link between specific surface reactions of NO_2_ at coinage metal-LC interfaces on LC ordering transitions, offering a new paradigm for harnessing surface chemistry to drive the macroscale reconfiguration of LC-based soft materials.

## 2 Methods

### 2.1 Materials

4-cyano-4'-pentylbiphenyl (5CB) was purchased from Jiangsu Hecheng Advanced Materials Co., Ltd. (Jiangsu, China), and 4′-octyl-4-biphenylcarbonitrile (8CB) was obtained from Sigma-Aldrich (Milwaukee, WI). Titanium (99.999%) and gold (99.999%) were purchased from Advanced Materials (Spring Valley, NY). Fisher’s Finest glass slides were purchased from Fisher Scientific (Pittsburgh, PA). CuSO_4_, AgClO_4_, HClO_4_ (70 wt %), and H_2_SO_4_ (98 wt %) were purchased from Sigma-Aldrich (Milwaukee, WI). Absolute ethanol (anhydrous, 200 proof) was purchased from Pharmco-AAPER (Brookfield, CT). Silicon wafers were purchased from Silicon Sense (Nashua, NH). Nitrogen dioxide in nitrogen (purity of NO_2_ was specified by the manufacturer as 99.9%) at a concentration of 10 ppm and synthetic air (Ultra Zero grade) was obtained from Airgas (Radnor, PA) and used as received. All chemicals and solvents were of analytical reagent grade and were used as received without any further purification. All aqueous solutions used in this study were made from deionized water with a resistivity of at least 18.2 MΩ.

### 2.2 Preparation of Gold Substrates

Semitransparent films of gold with thicknesses of 200 Å were deposited onto piranha-cleaned glass slides (see details in Section 1 of the Supporting Information) using an electron-beam evaporator (VEC-3000-C manufactured by Tekvac Industries, Brentwood, NY). A layer of titanium with a thickness of 20 Å was employed to enhance adhesion between the glass microscope slides and the gold films. The deposition rate of both gold and titanium was 0.2 Å s^–1^. The pressure in the evaporator was kept below 3 × 10^–6^ Torr throughout the deposition process. As observed by Chidsey et al., by using X-ray diffractometry, the predominant crystallographic face of polycrystalline Au deposited under vacuum is Au(111).^24^

### 2.3 Preparation of Silver and Copper Surfaces

Electrochemical experiments were performed using a Pine Instruments AFCBP1 bipotentiostat (Grove City, PA). The electrochemical cell was assembled in a standard three-electrode configuration using a gold film as the working electrode, a platinum wire mesh as the counter electrode, and a silver chloride electrode as the reference electrode (BASi, West Lafayette, IN). Ag overlayers on Au were prepared via electrodeposition from a solution containing 0.1 M HClO_4_ + 1 mM AgClO_4_.^25^ Cu overlayers on Au were prepared by electrodeposition from a 0.05 M H_2_SO_4_ + 1 mM CuSO_4_ solution. Cyclic voltammetry (CV) measurements (Figure S1a) confirmed that the Ag underpotential and bulk deposition occurred at 438 and 399 mV relative to the AgCl reference electrode. Similarly, for Cu (Figure S1b), two underpotential deposition regions were confirmed beginning at 250 and 52 mV (relative to the AgCl reference electrode), whereas bulk deposition began at 30 mV.^26^ For submonolayer deposition of Ag and Cu, we used 410 and 40 mV, respectively, and observed that the current density diminished over time to zero, consistent with an underpotential deposition (Figure S1c,d). The amount of Ag or Cu deposited onto the Au surfaces was controlled by the charge passed. One monolayer (ML) equivalent was defined as 220 μC cm^–2^ for Ag or 440 μC cm^–2^ for Cu on Au(111).^26^ After deposition, each electrode was removed from the electrochemical cell and rinsed for 2 min with flowing Milli-Q water.

### 2.4 X-ray Photoelectron Spectroscopy (XPS)

A Scienta Omicron ESCA-2SR was utilized to analyze samples under an operating pressure of approximately 10^–9^ Torr. Monochromatic Al Kα X-rays (1486.6 eV) were used, and photoelectrons were collected from a 5 mm diameter analysis area. The photoelectrons were collected at a 0° emission angle, with a source-to-analyzer angle of 54.7°. Survey scans were performed with a pass energy of 200 eV, while high-resolution scans were conducted using a 50 eV pass energy. The samples were conductive, and no charge neutralization was necessary. All scans were collected at 200 ms dwell times. All XPS results presented in this study were analyzed by CasaXPS software. A Gaussian–Lorentzian function was assumed for the oxygen and nitrogen components, while an asymmetrical Voigt function was utilized for metal components. This approach was guided by the results of prior studies.^17,19^

### 2.5 Fourier Transform Polarization-Modulation Infrared Reflectance Absorbance Spectroscopy (PM-IRRAS)

For all PM-IRRAS measurements, 100 μL of 2 mM 8CB in ethanol was spin-coated onto each metal-coated silicon wafer at 3000 rpm for 30 s. IR spectra of 8CB films deposited onto metal-coated silicon wafers were obtained using a Nicolet Magna-IR 860 FT-IR spectrometer, which employed a photoelastic modulator (PEM-90, Hinds Instruments, Hillsboro, OR), a synchronous sampling demodulator (SSD-100, GWC Technologies, Madison, WI), and a liquid N_2_-cooled mercury cadmium telluride (MCT) detector. All spectra (1000–4000 cm^–1^) were recorded at an incident angle of 83° with the modulation centered at either 2200 or 1500 cm^–1^. For each sample, 1000 scans were taken at a resolution of 4 cm^–1^. Data were collected as differential reflectance versus wavenumber, and the spectra were normalized and converted to absorbance units. Additional characterization of the as-deposited Au, Ag, and Cu surfaces by PM-IRRAS and XPS is provided in Figures S2–S4.

### 2.6 Preparation of Micrometer-Thick Films of LC With Free Surfaces

Copper-coated transmission electron microscopy (TEM) grids with a thickness of 20 μm (Electron Microscopy Sciences, Hatfield, PA) were placed onto the surface of a semitransparent metal film. The TEM grids defined square wells with lateral dimensions of 285 μm. 0.2 μL of LC was added to each TEM grid using a glass capillary, and excess LC was removed by wicking it into an empty capillary tube.

### 2.7 Preparation of LC Optical Cells

To fabricate the LC optical cells, two metal-coated glass slides were aligned facing each other and separated by a glass spacer with a diameter of 5 μm. A volume of 2 μL of 5CB, heated to form an isotropic phase (35 °C < *T* < 40 °C), was drawn into the cavity between the two surfaces of the optical cell via capillarity. The optical appearance of the LC film was characterized by using an Olympus BX-60 polarized light microscope in transmission mode (Olympus, Japan). To perform conoscopic imaging of the LC films, a Bertran lens was inserted into the optical path.^27^

### 2.8 Anchoring Transitions of LCs Induced by NO_2_

LC samples hosted within TEM grids supported on metal films, prepared as described above, were exposed to a stream of N_2_ containing 10 ppm of NO_2_ using a flow cell that permitted observation of the LC with a polarized light microscope (CH40, Olympus, Melville, NY). A previous publication contains a detailed description of the flow cell.^28^ The flow rate of gas through the flow cell was maintained at 1000 mL min^–1^ using a series of rotameters (Aalborg Instruments and Controls, Inc., Orangeburg, NY). Optical micrographs of LC films in the flow cell are shown in Figures S5–S7.

### 2.9 Measurement of Optical Retardance

The optical retardance values (ΔΓ) of LC films were measured using two methods. First, retardance values were estimated by matching the interference colors of LC samples, imaged between crossed polarizers using white light, to the Michel-Levy color chart. Second, quantitative measurements were obtained using a Berek compensator (Olympus, Melville, NY). For each sample, measurements were taken at five locations and were averaged. The tilt angle of the LC (θ, measured from the surface normal) was calculated from the retardance using

1 $$\Delta \Gamma \approx \int_{0}^{d}(\frac{n_{o}n_{e}}{\sqrt{n_{o}^{2}\sin^{2}\left(\left(1-\frac{z}{d}\right)\theta_{\mathrm{bottom}}\right)+n_{e}^{2}\cos^{2}\left(\left(1-\frac{z}{d}\right)\theta_{\mathrm{bottom}}\right)}}-n_{o})dz$$

where *n*_o_ and *n*_e_ are the indices of refraction perpendicular and parallel to the optical axis of the LC, respectively, and *d* is the thickness of the LC film in the *z*-direction. The solution to eq 1 yields θ_bottom_, the tilt angle of the LC at the bottom substrate (measured from the surface normal).

### 2.10 Density Functional Theory (DFT) Calculations

Density functional theory calculations were performed using the Vienna *Ab initio* Simulation Package (VASP).^29,30^ The exchange–correlation was modeled using the Perdew–Burke–Ernzerhof generalized gradient approximation (GGA-PBE), with a plane-wave cutoff energy of 400 eV.^31^ Electron–core interactions were described using projector-augmented wave (PAW) potentials.^32,33^ The adsorption of 5CB on Au, Ag, and Cu surfaces was modeled using a 4-cyanobiphenyl (PhPhCN) surrogate molecule following methods established in our prior work.^4,5^ Additional information regarding the choice of PhPhCN as a computational surrogate for 5CB is provided in the Supporting Information. Dispersion interactions were modeled using Grimme’s D3 dispersion correction scheme with zero damping.^34^ This combination of functional and dispersion treatment has been previously validated by accurately reproducing the experimental anchoring behavior of 5CB on Au-containing films^4^ and provides close agreement between DFT-predicted and experimental XPS spectra for Ag surfaces,^35^ which together account for two-thirds of the metal substrates studied. To maintain methodological consistency, the same PBE-D3 approach was applied to Cu-based systems.

All surfaces were described using (4 × 4) supercells comprised of four atomic layers, in which the bottommost two layers were fixed at their bulk lattice spacings. To model both the predominant facet and defects/undercoordinated sites of the deposited Au film, we used Au(111) and Au(874), respectively. The Ag surface was modeled as *p*(4 × 4)-O/Ag(111) with a single Ag vacancy, which is composed of a reconstructed Ag–O overlayer supported on four layers of unreconstructed Ag(111), and has been identified as the likely surface state of Ag films exposed to NO_2_ under ambient conditions.^36,37^ Integration of the Brillouin zone was performed using a (4 × 4 × 1) Γ-centered Monkhorst–Pack *k*-point mesh for all surface unit cells.^38^ A convergence criterion was adopted for differences in energy and forces acting on each atom between electronic steps, less than 10^–6^ eV and 0.01 eV/Å, respectively. Vibrational frequencies were obtained using second-order finite differences with a step size of 0.007 Å for numerical differentiation of the mass-weighted Hessian matrix. All structures were visualized using OVITO or VESTA.^39,40^

To reflect the changing oxidation state of the Cu surface in response to NO_2_ (see Section 3.4), we modeled the ambient-exposed (that is, pre-NO_2_ exposure) surface as Cu_2_O­(111), whereas the post-NO_2_ exposure surface was modeled as CuO(111). These bulk oxides serve as simplified models for probing the influence of the surface oxidation state on LC anchoring. The rationale for modeling both the pre- and post-NO_2_ exposure Cu surfaces as bulk oxides reflects the limited consensus on the precise atomic structure of Cu surface oxides under reactive conditions.^41^ DFT + *U* corrections were applied to all calculations involving Cu_2_O and CuO. For Cu_2_O (Cu in the 1+ oxidation state), an on-site Coulomb correction of *U* = 5.2 eV was applied to the Cu d states. This value has been reported to accurately reproduce the experimental XPS band features of Cu_2_O.^42^ For CuO, however, reflecting the higher 2+ oxidation state, a larger on-site correction of *U* = 7 eV, which provides better agreement between calculated and experimental values for the lattice parameters, magnetic properties, and band gaps of CuO,^43,44^ was applied to the Cu d states. Consistent with prior studies, we find that Cu_2_O­(111) preferentially adopts a reconstructed overlayer in which coordinatively unsaturated Cu atoms relax toward adjacent Cu atoms, resulting in a pronounced vacancy-like gap within the surface layer.^45–47^ A comparison of DFT-calculated structural parameters to experimental values is shown in Table S1. Structural representations of the Au, Ag, and Cu surfaces used to model LC-surface and NO_2_-surface interactions are shown in Figure 1.

**Figure 1:**
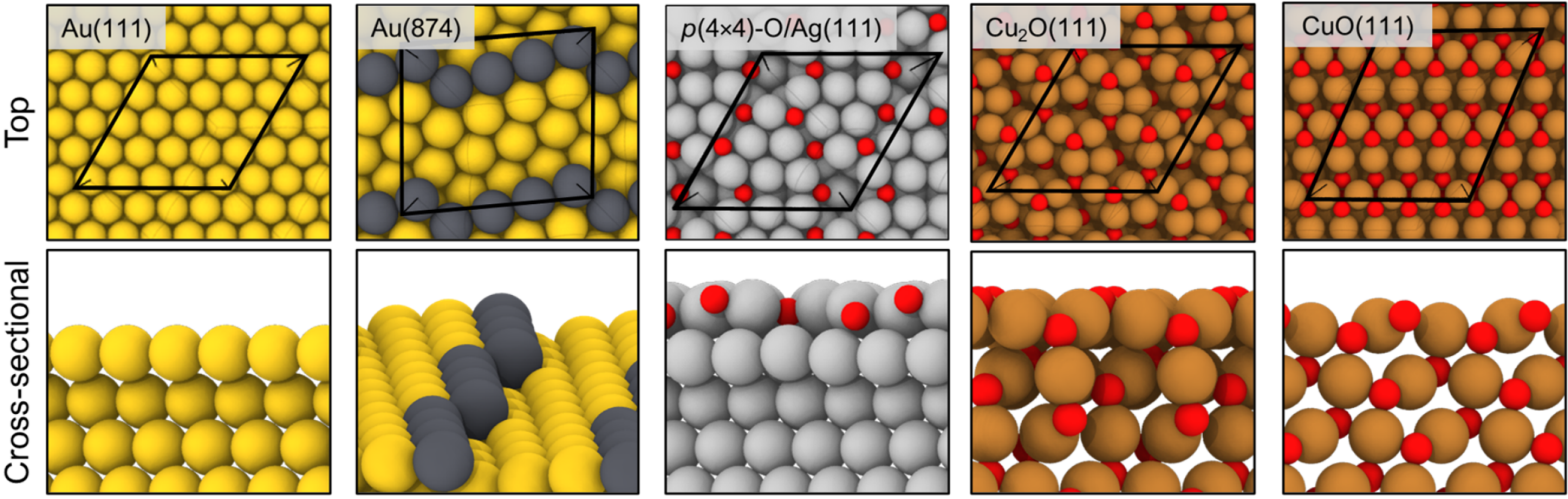
Top and cross-sectional views of Au(111), Au(874), *p*(4 × 4)-O/Ag(111), Cu_2_O­(111), and CuO(111) slabs used to model surface interactions with 5CB surrogates and NO_2_. The following atomic color scheme was adopted: gold (Au terrace), dark gray (Au step), gray (Ag), bronze (Cu), and red (O). The surface unit cell is denoted by black lines.

Adsorption free energies normalized per unit area for PhPhCN (Δ*G*_ads_^LC^) were calculated as

2 $$\Delta G_{\mathrm{ads}}^{\mathrm{LC}}=\frac{1}{A}\left[G_{\mathrm{slab}+\mathrm{PhPhCN}}-G_{\mathrm{slab}}-n_{\mathrm{PhPhCN}}\mu_{\mathrm{PhPhCN}}\right]$$

where *G*_slab+PhPhCN_ is the free energy of PhPhCN adsorbed to the slab, *G*_slab_ is the free energy of the slab (pristine or decorated by NO_2_, N_2_O_4_, and NO_3_), *n*_PhPhCN_ is the number of PhPhCN molecules per unit cell, and μ_PhPhCN_ is the DFT-calculated chemical potential of PhPhCN in the gas phase at 298.15 K and 1 atm. The chemical potential of PhPhCN (1 atm) was calculated using

3 $$\mu_{\mathrm{PhPhCN}}=E_{\mathrm{PhPhCN}}+\Delta \mathrm{ZPE}+\int_{0}^{T}C_{P}\left(T\right)\mathrm{dT}-\mathrm{TS}\left(T\right)$$

Here, *E*_PhPhCN_ is the DFT-calculated electronic energy of PhPhCN, ΔZPE is the zero-point energy correction, *C*_P_ is the heat capacity, *T* is the temperature (298.15 K), and *S* is the entropy. More negative values of Δ*G*_ads_^LC^ denote stronger binding, whereas more positive Δ*G*_ads_^LC^ values denote weaker binding. Entropic and enthalpic contributions to the free energies of gas-phase PhPhCN were calculated using the three-dimensional ideal gas approximation. Entropic contributions to the free energy of surface-bound species were estimated using the harmonic oscillator approximation for PhPhCN, NO_2_, and NO_3_. For each surface-adsorbed N_2_O_4_, a weakly physisorbed species, we instead approximate the entropic contribution as one-third of the experimental gas-phase entropy of two NO_2_ molecules,^48^ consistent with the approach adopted in our previous work.^35^

The adsorption of PhPhCN in a planar orientation on Au(111) and *p*(4 × 4)-O/Ag(111) was studied at a coverage of 2/16 ML, corresponding to saturation of the unit cell surface area. In the perpendicular orientation, a coverage of 5/16 ML of PhPhCN was used for Au(111) and *p*(4 × 4)-O/Ag(111) surfaces, representing the coverage that yields the lowest adsorption free energy per unit area (Figure S8). Because of the expanded lattice parameters of copper oxide surfaces, we employ higher coverages of PhPhCN for Cu_2_O­(111) and CuO(111): (1) 4/16 ML PhPhCN for a planar orientation and (2) 6/16 ML PhPhCN for a homeotropic orientation. Additional information on coverage selection for the Cu surfaces can be found in Section 4 of the Supporting Information.

The strength of electrostatic interactions between adsorbates on Au(111) was evaluated by calculating the overall electrostatic interaction energy per unit cell (Δ*E*_int,A‑B_^INN^) between first nearest-neighbor (INN) NO_2_–PhPhCN and NO_2_–NO_2_ pairs using

$$\Delta E_{\mathrm{int},A‐B}^{INN}=\underset{i}{\sum }{\left(\Delta E_{\mathrm{int},A‐B}^{INN}\right)}_{i}$$ 4

5 $$\left({\Delta E}_{\mathrm{int},A‐B}^{\mathrm{INN}}\right){\;}_{i}=\frac{\mu_{z,A}\mu_{z,B}}{4\pi \varepsilon_{0}r^{3}}$$

where μ_z,A_ and μ_z,B_ are the *z*-component (surface normal) dipole moments of isolated NO_2_ or PhPhCN molecules adsorbed onto Au(111), with the sign chosen such that positive values correspond to charge accumulation toward the surface and negative values to charge accumulation toward the vacuum layer, *ε*_0_ is the vacuum permittivity, and *r* is the Euclidean separation distance between species. As the dipole of perpendicularly bound PhPhCN lies almost entirely in the direction of the surface normal, we consider only the *z*-component of adsorbate dipoles when calculating Δ*E*_int,A‑B_^INN^. Adsorbate dipoles were calculated at their equilibrium position using

6 $$\mu_{z,i}=\int_{V}z\rho \left(z\right)\mathrm{dV}$$

where *z* is the *z*-coordinate of the charge density normalized to the center of charge of the unit cell, ρ is the charge density, and *V* is the volume of the unit cell. Additional information on the applied scheme for calculating Δ*E*_int,A‑B_^INN^ can be found in Scheme S1. For each NO_2_ coverage, the stabilization gained by coadsorbing NO_2_ with perpendicularly bound PhPhCN (ΔΓ_diff_^LC^) is defined as

7 $${\Delta \Gamma }_{\mathrm{diff}}^{\mathrm{LC}}=A\left({\Delta G}_{\mathrm{ads},i}^{\mathrm{LC}}-{\Delta G}_{\mathrm{ads},0}^{\mathrm{LC}}\right)$$

where Δ*G*_ads,i_^LC^ and Δ*G*_ads,0_^LC^ are the adsorption free energies per unit area for homeotropic PhPhCN coadsorbed with *i* molecules of NO_2_ and perpendicular PhPhCN adsorbed on the pristine metal surface, respectively. The energetic penalty associated with displacing NO_2_ upon coadsorption with PhPhCN (Δ*E*_NO_2__) was calculated through:

8 $$\Delta E_{NO_{2}}=E_{\mathrm{slab}+NO_{2}}^{\mathrm{single}‐\mathrm{point}}-E_{\mathrm{slab}+NO_{2}}^{\mathrm{relaxed}}$$

Here, *E*_slab+NO_2__^single‑point^ represents the energy of NO_2_ adsorbed on Au(111) at positions constrained to those observed under coadsorption with 5/16 ML PhPhCN but recalculated in the absence of PhPhCN via single-point energy calculations. *E*_slab+NO_2__^relaxed^ corresponds to the fully relaxed minimum-energy configuration of NO_2_ in the absence of PhPhCN on the same surface at the identical coverage. The resulting energy difference isolates the destabilization imposed by PhPhCN on NO_2_ adsorption to the Au surface.

## 3 Results and Discussion

### 3.1 Fabrication and Characterization of Coinage Metal Films

Coinage metal films were fabricated by depositing 20 nm of Au onto glass substrates via electron-beam deposition at an incidence angle of 10 ± 3° relative to substrate normal. XPS measurements (Figures S2 and S3) confirmed the presence of metallic Au, evidenced by the Au 4f_7/2_ peak at 83.9 eV.^49^ Weak C 1s (284.5 eV) and O 1s (531.9 eV) signals measured on the Au substrate suggested the presence of adventitious hydrocarbons, commonly adsorbed from ambient exposure.^50^ Comparable C 1s features were observed on Ag and Cu films deposited on Au, independent of overlayer thickness (Figure S3b,d). As Au surfaces are resistant to oxidation even at elevated temperatures (500 K) and O_2_ partial pressures (1.8 atm),^49,51^ the O 1s feature likely originates from adventitious oxygen-containing hydrocarbons, rather than oxidation of the Au film. To quantify the surface-bound hydrocarbon species, PM-IRRAS measurements, offering greater surface sensitivity than XPS, were performed on bare Au films and on Au prefunctionalized with a monolayer of a representative hydrocarbon species, 1-hexadecanethiol (HDT; Figure S4). While HDT-coated samples displayed intense vibrational bands associated with the C–H stretching region, the as-deposited Au films showed only weak signals, consistent with minimal adventitious hydrocarbon adsorption.

Unlike Au, which is inert to O_2_ dissociation at room temperature, both Ag and Cu readily form surface oxides when exposed to oxygen under ambient conditions.^52,53^ To determine whether this oxidation is limited to the near-surface region or extends into the metal bulk, Ag and Cu films of varying thicknesses were electrodeposited onto electron-beam-fabricated Au substrates (see Section 2 for details). XPS of a 1.13-ML-thick Ag film (Figure 2a,b) revealed clear signatures of surface oxidation, evidenced by Ag 3d_5/2_ (367.8 eV) and O 1s (530.4 and 531.9 eV) features.^54–58^ As established in prior studies, the O 1s signal at 530.4 eV corresponds to chemisorbed oxygen on Ag, commonly attributed to surface hydroxyl groups,^57,58^ while the broader 531.9 eV feature is consistent with adventitious hydrocarbons.^59^ The observed O 1s (530.4 eV) to Ag 3d_5/2_ (367.8) ratio of 0.35 aligns with previously reported values of 0.38 ^60^ and 0.41,^58,61^ supporting the formation of a reconstructed Ag surface oxide (*p*(4 × 4)-O/Ag(111) reconstruction shown in Figure 1).^36,37^ As the Ag film thickness was increased from 0.23 to 25 ML of Ag, the Ag 3d_5/2_ binding energy shifted from 367.8 to 368.2 eV (Figure 2a), approaching the value of metallic Ag.^54–56^ Notably, the O 1s intensity reached a maximum at 1.13 ML of deposited Ag and remained constant thereafter, indicating that Ag oxidation was confined to the near-surface region (Figure 2b).

**Figure 2:**
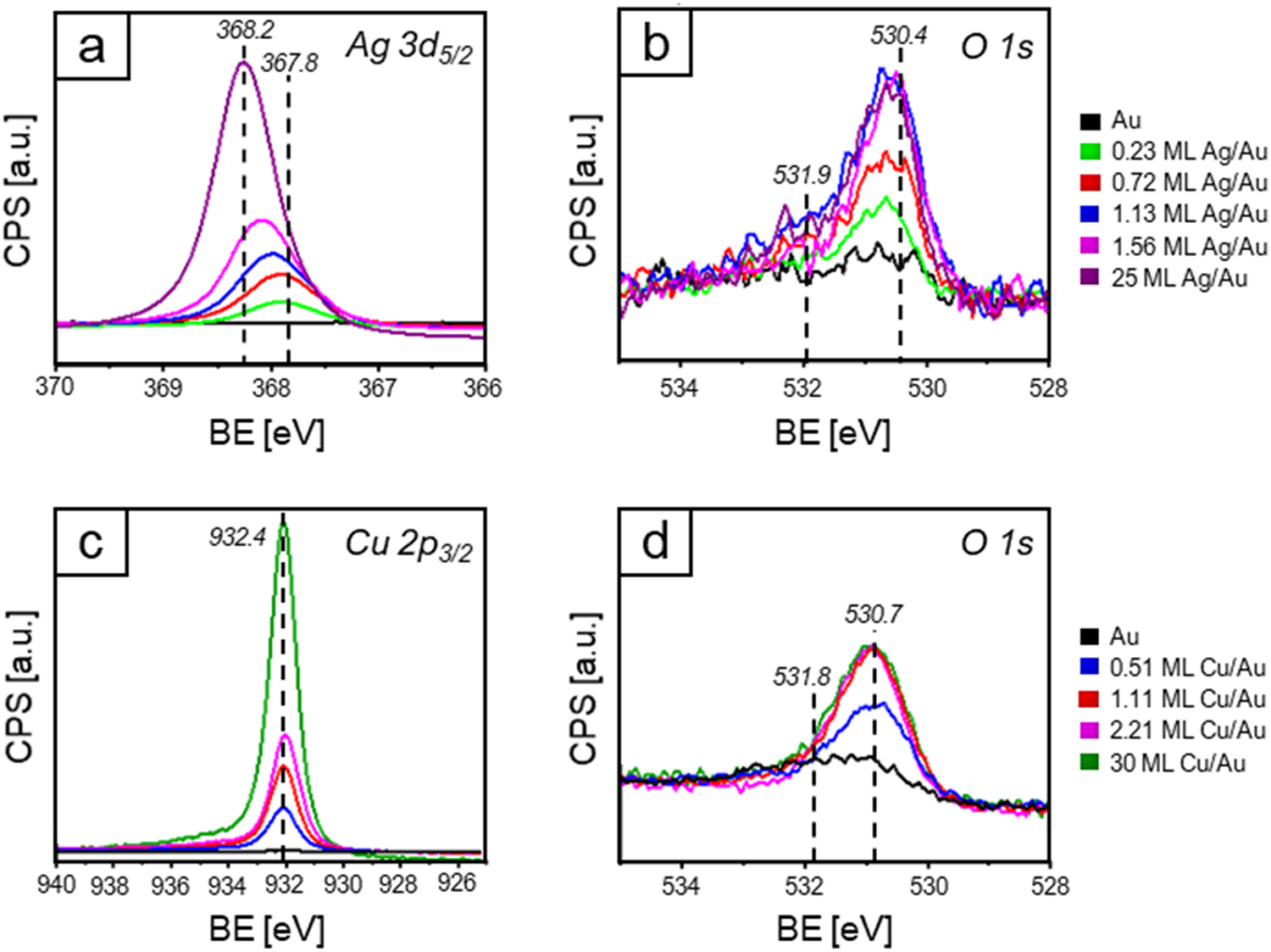
X-ray photoelectron spectra of Ag and Cu films of varying thickness deposited on 20-nm-thick Au films. (a) Ag 3d_5/2_ spectra of Ag/Au films, (b) O 1s spectra of Ag/Au films, (c) Cu 2p_3/2_ of Cu/Au films, and (d) O 1s of Cu/Au films.

XPS analysis of a 1.11-ML-thick Cu/Au film (Figure 2c,d) revealed a Cu 2p_3/2_ peak at 932.4 eV, consistent with both metallic Cu and Cu_2_O bulk oxides.^62^ Deconvolution of the O 1s spectrum yielded components at 530.7 and 531.8 eV, attributed to chemisorbed oxygen on Cu (531 eV)^63^ and oxygen present in adventitious oxygen-containing species (531.9 eV),^50,59^ respectively. Although the overlapping Cu 2p_3/2_ binding energies of Cu^0^ (932.6 eV) and Cu^+^ (932.4 eV) complicate precise phase identification,^62^ the O 1s (530.7 eV) to Cu 2p_3/2_ (932.4 eV) intensity ratio of ∼0.48 is consistent with a Cu_2_O-like surface structure.^58^ As the Cu film thickness increased from 1.11 to 30 ML of Cu, the Cu 2p_3/2_ peak intensity at 932.4 eV grew proportionately, while the O 1s signal at 530.7 eV saturated at the value obtained for 1.11 ML Cu/Au for thicker Cu films. This result, mirroring the behavior observed for Ag films, indicates that oxidation to form Cu_2_O was confined to the near-surface region of the Cu film.

### 3.2 Response of 5CB to NO_2_ over Gold Surfaces

With the surface structures of the as-deposited Au, Ag, and Cu films characterized, we studied the orientational response of 5CB to the NO_2_ exposure. We aimed to elucidate the physical factors governing the orientational response of 5CB on the coinage metal surfaces rather than to provide a complete characterization of the surface state. To isolate the intrinsic reactivity of each coinage metal surface, all subsequent measurements are focused on Ag and Cu films of sufficient thickness to minimize the effects imposed by the underlying Au substrate on LC anchoring.

To investigate how NO_2_ adsorption modulates the interaction between 5CB and Au surfaces, we exposed micrometer-thick films of 5CB supported on Au films dominated by (111) domains to a stream of N_2_ containing 10 ppm of NO_2_. Prior to NO_2_ exposure, polarized optical microscopy in transmission mode revealed a green and pink birefringent texture of the nematic 5CB film (Figure 3a), with a measured retardance of 1710 ± 31 nm, corresponding to a nearly planar orientation of 5CB at the Au interface (tilt angle of 88 ± 1° relative to the surface normal). This anchoring state is consistent with our previous results showing planar anchoring of 5CB on Au surfaces.^4,64^ DFT calculations (Figure 3d) for the binding of 4-cyanobiphenyl (PhPhCN), the computational surrogate used for 5CB, further show that PhPhCN adsorbs preferentially to pristine Au(111) (θ_NO_2__ = 0/16 ML) in a planar orientation (Δ*G*_ads_^LC^ = −1.51 eV nm^–2^). Adsorption of PhPhCN in a homeotropic orientation was comparatively weaker (Δ*G*_ads_^LC^ = −1.27 eV nm^–2^). We also observed that the azimuthal orientation of 5CB was degenerate as the cross-polarizers were rotated (Figure S5), consistent with previous work.^6^ Although Au was deposited with an incidence angle (measured from the surface normal) of 10 ± 3°, this angle was not large enough to induce a uniform azimuthal orientation of LCs at the Au-LC interface. Previous work has reported that an incidence angle larger than 30° is necessary to induce a uniform in-plane alignment of nematic 5CB.^64^

**Figure 3:**
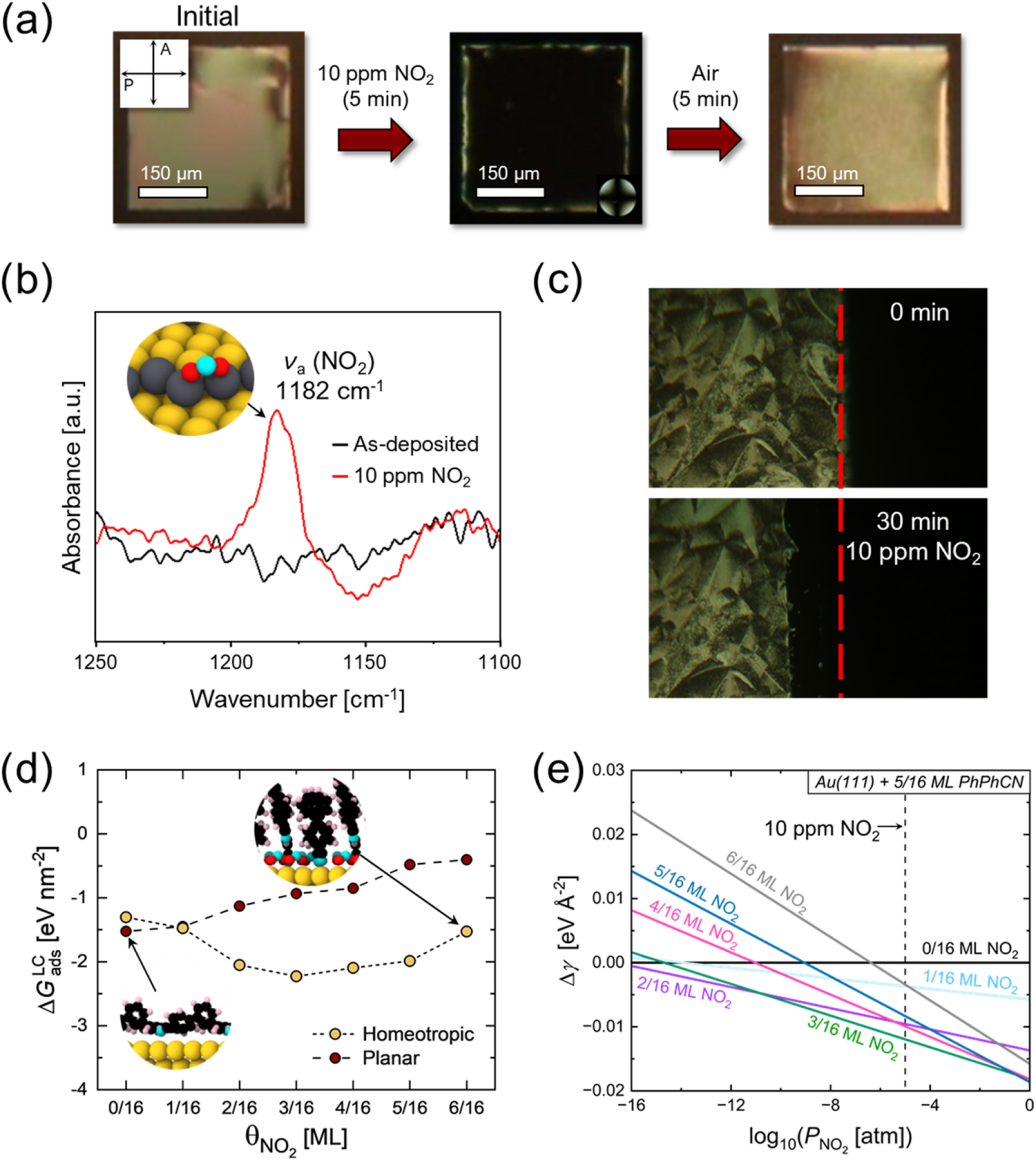
(a) Optical micrographs (crossed polars; top view) showing 20-μm-thick films of 5CB supported on Au films, which were exposed to 10 ppm of NO_2_ for 5 min and then air for another 5 min. (b) PM-IRRAS for the as-deposited electron-beam-deposited Au surface prior to NO_2_ exposure (black trace) and upon exposure to 10 ppm of NO_2_ at room temperature (red trace). The inset illustrates the DFT-calculated adsorption mode of 1/16 ML NO_2_ on Au(874) kink sites. (c) Optical image of an LC optical cell made from two Au substrates and filled with 5CB, which was exposed to NO_2_ for 30 min. The red dashed line indicates the edge of the optical cell that was exposed to NO_2_. (d) Calculated PhPhCN adsorption free energy normalized per unit area (Δ*G*_ads_^LC^) on Au(111) surfaces with varying coverages (θ_NO_2__) of NO_2_ at 298.15 K. (e) Calculated surface free energy (Δγ) of Au(111) decorated with 5/16 ML (perpendicular) PhPhCN as a function of NO_2_ coverage (1/16 to 6/16 ML) at 298.15 K. The horizontal black line denotes the 5/16 ML PhPhCN-decorated Au(111) surface in the absence of adsorbed NO_2_. The following atomic color scheme was adopted: H (pink), C (black), O (red), N (cyan), Au terrace (gold), and Au step (dark gray).

Upon exposure to NO_2_, the 5CB film supported on Au underwent a continuous optical transition from bright to dark (Figure 3a), consistent with a shift from planar to perpendicular alignment of 5CB at the Au interface. This response is similar to one observed in a prior study of the response of an E7 LC mixture to NO_2_ on Au.^65^ PM-IRRAS measurements on the electron-beam-deposited Au surface exposed to 10 ppm of NO_2_ in the absence of 5CB (Figure 3b) showed the emergence of a band at 1182 cm^–1^ attributable to the symmetric stretch of surface-bound NO_2_ in an (O,O’)-chelating adsorption geometry.^15,17,66,67^ We propose that this IR feature reflects adsorption of NO_2_ at Au defect sites, as prior TPD studies show that NO_2_ desorbs from Au(111) terraces below room temperature but persists on defects up to ∼300 K.^15^ In the presence of 5CB surrogates, DFT calculations indicate that 5/16 ML of PhPhCN coadsorbed in a perpendicular orientation stabilizes NO_2_ adsorption on Au(111) terraces, yielding an adsorption free energy of −0.66 eV at 298.15 K and 10 ppm of NO_2_. This value is comparable to that of NO_2_ bound to Au(874) defects (−0.58 eV) and significantly enhanced compared to NO_2_ adsorption on pristine Au(111) (−0.46 eV). Together with the prior TPD results, these calculations suggest that, in the presence of 5CB, NO_2_ binds to Au(111) with a strength similar to that at defect sites of the clean polycrystalline surface Au.^15^ When coadsorbed with 5CB, NO_2_ is therefore expected to adsorb stably and accumulate on Au(111) facets that dominate the 5CB-Au interface. These adsorbed NO_2_ species are responsible for the observed reorientation of 5CB in response to NO_2_.

To verify that the reorientation of 5CB in Figure 3a reflects a change in the lowest free energy orientation of the LC at the Au surface (i.e., a change in the so-called easy axis), as opposed to a weakening of the anchoring energy at the Au-LC interface that allows reorientation of the LC under the influence of the LC-gas interface,^68^ additional experiments were performed using 5CB films deposited between two Au surfaces separated by 4-μm-thick spacers in a sandwich arrangement (Figure 3c). Following exposure to NO_2_, we observed a change in the orientation of 5CB to progress from the edge of the LC cell toward the center, a change that is consistent with diffusion of NO_2_ into the LC and an alignment change of 5CB that is triggered by the reorientation of the easy axis of the LC supported on the Au surface (and not an orientation driven by the LC-air interface). We then examined the reversibility of the NO_2_-induced anchoring transition of 5CB. Following exposure of the 5CB film to 10 ppm of NO_2_, we subsequently introduced a stream of synthetic air (20% O_2_ in N_2_) into the LC flow cell for 5 min. Treatment with synthetic air induced a dark (homeotropic) to bright (planar) transition in the optical appearance of the 5CB film (Figure 3a) and a recovery of the planar anchoring of 5CB on Au observed prior to NO_2_ exposure. Control experiments, where Au surfaces were sequentially exposed to NO_2_ and then air prior to LC deposition, further yielded planar alignment of 5CB (Figure S5b).

To gain atomistic insight into the observed planar-to-perpendicular transition of 5CB supported on Au surfaces following exposure to NO_2_, we conducted DFT calculations to assess the binding strength of PhPhCN on Au(111) surfaces decorated with varying NO_2_ coverages. Other surface species, such as N_2_O_4_ and N_2_O_3_, were not considered, as they are known to desorb or decompose on Au at temperatures above 200 K and were not detected experimentally in our PM-IRRAS spectra (Figure 3b).^15,67^ Similarly, NO_2_ dissociation products (NO, O, and N) were excluded, consistent with prior studies showing that NO_2_ does not dissociate on Au under ambient experimental conditions.^15^

As shown in Figure 3d, increasing NO_2_ coverage destabilizes adsorption of PhPhCN in a parallel binding mode while enhancing perpendicular binding. Notably, adsorption of PhPhCN in a perpendicular orientation becomes energetically preferred over the parallel binding mode beyond coverages of 1/16 ML NO_2_*. To estimate the equilibrium NO_2_ coverage of the Au surface under the experimental conditions of Figure 3a, we constructed an *ab initio* phase diagram for the adsorption of between 1/16 and 6/16 ML of NO_2_ on 5/16 ML PhPhCN-decorated (perpendicular) Au(111) (Figure 3e). At room temperature and 10 ppm of NO_2_, the thermodynamically favored state of the LC-decorated Au surface corresponds to 3/16 ML NO_2_* (Figure 3e), which is comparatively lower than the NO_2_ saturation coverage (0.4 ML) observed by Bartram and Koel on pristine Au(111) at 100 K.^67^ At 3/16 ML NO_2_*, the calculated adsorption free energy of PhPhCN strongly favors perpendicular binding (Δ*G*_ads_^LC^ = −2.20 eV nm^–2^, Figure 3d) over planar anchoring (Δ*G*_ads_^LC^ = −0.92 eV nm^–2^, Figure 3d). These theoretical predictions qualitatively align with the experimental anchoring transitions observed in Figure 3a, suggesting that NO_2_ promotes perpendicular alignment of 5CB through favorable interfacial interactions with adsorbed NO_2_ species.

To elucidate the origin of the stabilizing interactions between NO_2_ and PhPhCN adsorbed in a perpendicular orientation on Au(111), we calculated the net surface-normal dipoles of NO_2_ and PhPhCN adsorbed to Au(111). In the absence of PhPhCN, NO_2_ adsorption on Au(111) generates a net surface-normal dipole moment of +0.30 e^–^Å per NO_2_ molecule (1/16 ML NO_2_), with net electron density oriented toward the center of charge of the slab. By contrast, PhPhCN adsorbed in a perpendicular orientation on Au(111) produces an oppositely oriented dipole moment of ca. −1.10 e^–^Å per PhPhCN molecule (average), evaluated for a perpendicularly aligned 5/16 ML PhPhCN overlayer obtained upon coadsorption with 3/16 ML NO_2_. This suggests favorable dipole–dipole interactions between perpendicular PhPhCN and coadsorbed NO_2_. PhPhCN adsorbed in a parallel orientation, however, yields a weak surface-normal dipole moment of ca. +0.05 e^–^Å per PhPhCN molecule (average), calculated for a parallel aligned 2/16 ML PhPhCN overlayer upon coadsorption with 3/16 ML NO_2_.

Previous studies have demonstrated that antiparallel dipoles between coadsorbed species can significantly stabilize otherwise unfavorable adsorbed states on metal surfaces.^69,70^ For example, on Pt(111), high CO coverage induces a mixed atop–bridge site arrangement, stabilized by electrostatic interactions between anti-aligned dipoles between adjacent CO molecules, despite atop-only adsorption being preferred in vacuum.^70^ Motivated by these findings, we computed the first nearest-neighbor electrostatic interaction energy (see Section 2) between perpendicular PhPhCN and coadsorbed NO_2_ molecules (Δ*E*_int,PhPhCN–NO_2__^INN^), as well as NO_2_–NO_2_ pairs (Δ*E*_int,NO_2_–NO_2__^INN^), across the adsorption configurations reported in Figure 3d to quantitatively assess whether the trends in PhPhCN binding are driven by electrostatic interactions with NO_2_.

To account for energy penalties associated with displacing NO_2_ from its preferred adsorption geometry on pristine Au(111), the slab reference state used to compute Δ*G*_ads_^LC^ in Figure 3d, we introduced a correction term that quantifies the energetic cost of perturbing NO_2_ upon coadsorption with PhPhCN (Δ*E*_NO_2__; gray bars, Figure 4). The sum of Δ*E*_int,PhPhCN‑NO_2__^INN^, Δ*E*_int,NO_2_–NO_2__^INN^, and Δ*E*_NO_2__ results in a combined contribution (white bars, Figure 4) that qualitatively reproduces the observed stabilization of perpendicular PhPhCN in the presence of NO_2_ (ΔΓ_diff_^LC^; black line, Figure 4), defined as the change in the perpendicular Δ*G*_ads_^LC^ for each coverage of NO_2_ relative to the 0/16 ML NO_2_ case in Figure 3d (see eq 7). These results, together with the binding energetics presented in Figure 3d, support our conclusion that the experimentally observed planar-to-perpendicular anchoring transition of 5CB upon NO_2_ exposure (Figure 3a) arises from favorable electrostatic interactions between coadsorbed NO_2_ and perpendicularly bound 5CB mesogens (Δ*E*_int,PhPhCN‑NO_2__^INN^). Moreover, stabilization of the perpendicular alignment of PhPhCN is accompanied by destabilization of the planar configuration (Figure 3d), consistent with our previous findings that surface adsorbates, such as oxygen on Pd films, disrupt planar π-bonding and dispersion interactions between the aromatic rings of 5CB and the metal substrate.^5^ We note that, while the decomposition in Figure 4 captures the dominant interactions governing how NO_2_ affects the binding of homeotropic PhPhCN on Au(111), an offset between ΔΓ_diff_^LC^ (black line) and Δ*E*_int,PhPhCN‑NO_2__^INN^ + Δ*E*_int,NO_2_–NO_2__^INN^ + Δ*E*_NO_2__ (white bars) indicates that other minor contributions may be present, but do not qualitatively alter the coverage-dependent trend.

**Figure 4:**
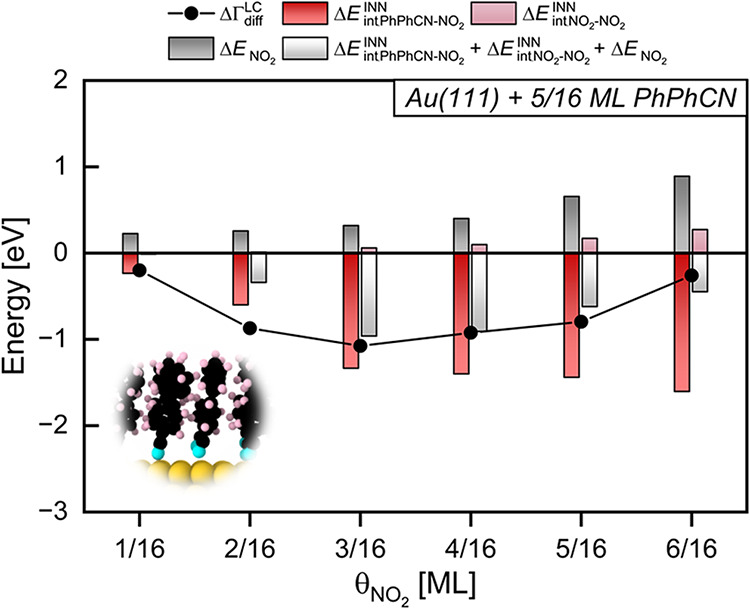
Interaction energy governing the homeotropic binding strength of PhPhCN as a function of NO_2_ coverage. The black line shows the change in adsorption free energy of 5/16 ML PhPhCN when the NO_2_ coverage is increased by 1/16 ML relative to the corresponding *x*-axis value (ΔΓ_diff_^LC^). Gray bars indicate destabilizing interactions that weaken NO_2_ binding to Au(111) upon coadsorption with 5/16 ML PhPhCN (Δ*E*_NO_2__). Red and pink bars represent first nearest-neighbor electrostatic interactions between homeotropic PhPhCN and coadsorbed NO_2_ molecules (Δ*E*_int,PhPhCN‑NO_2__^INN^) or NO_2_–NO_2_ pairs (Δ*E*_int,NO_2_–NO_2__^INN^), respectively. White bars correspond to the sum of red, pink, and gray contributions at each NO_2_ coverage. The inset displays 5/16 ML PhPhCN adsorbed to Au(111) in the absence of NO_2_. The following atomic color scheme was adopted: H (pink), C (black), O (red), N (cyan), and Au (gold).

### 3.3 Response of 5CB to NO_2_ over Silver Surfaces

We next examined the interaction of 5CB with NO_*x*_ species over oxygen-reconstructed Ag surfaces identified on the fabricated Ag/Au films in Figure 2, focusing on the model *p*(4 × 4)-O/Ag(111) surface structure (shown in Figure 1). Prior studies have established that NO_2_ can react with surface oxygen present in the oxide overlayers formed on ambient-exposed Ag surfaces.^19,21,22,35^ However, the identity of the resulting NO*_x_* species remains debated. Polzonetti et al. attributed the observed NO_*x*_ species to nitrate (NO_3_), based on a 1:3 intensity ratio between the N 1s and O 1s XPS features at binding energies of 405.8 and 531.8 eV, respectively.^21^ Yet, prior studies place the N 1s binding energy of NO_3_ closer to 407 eV,^16,37^ while nitrogen tetroxide (N_2_O_4_) yields an N 1s signature more consistent with experimental observations (405.4 eV).^37^ Moreover, the O 1s feature at 531.8 eV overlaps with common adventitious hydrocarbon species,^59^ complicating unambiguous identification of the formed NO_*x*_ species as NO_3_. Our recent XPS data and core-level shift calculations support the assignment of N_2_O_4_, rather than NO_3_, as the dominant nitrogen species on Ag films prepared under ambient conditions.^35^ However, we note that these prior experiments were performed in the absence of LCs, which may modify the energetics of NO_2_ adsorption or its conversion to N_2_O_4_/NO_3_.

Building from these prior studies, we exposed nematic 5CB supported on 30-ML-thick films of ambient-exposed Ag electrochemically deposited on Au to a nitrogen stream containing 10 ppm of NO_2_. In the absence of NO_2_, the Ag films induced an initial perpendicular alignment of 5CB, as evidenced by the extinction of transmitted light between crossed polars (Figure 5a). This observation of perpendicular anchoring of 5CB is in agreement with DFT calculations, which showed that PhPhCN preferentially adsorbs in a perpendicular configuration on pristine *p*(4 × 4)-O/Ag(111) (Δ*G*_ads_^LC^ = −2.86 eV nm^–2^; θ_NO_2__ = 0/16 ML in Figure 5b–d).

**Figure 5:**
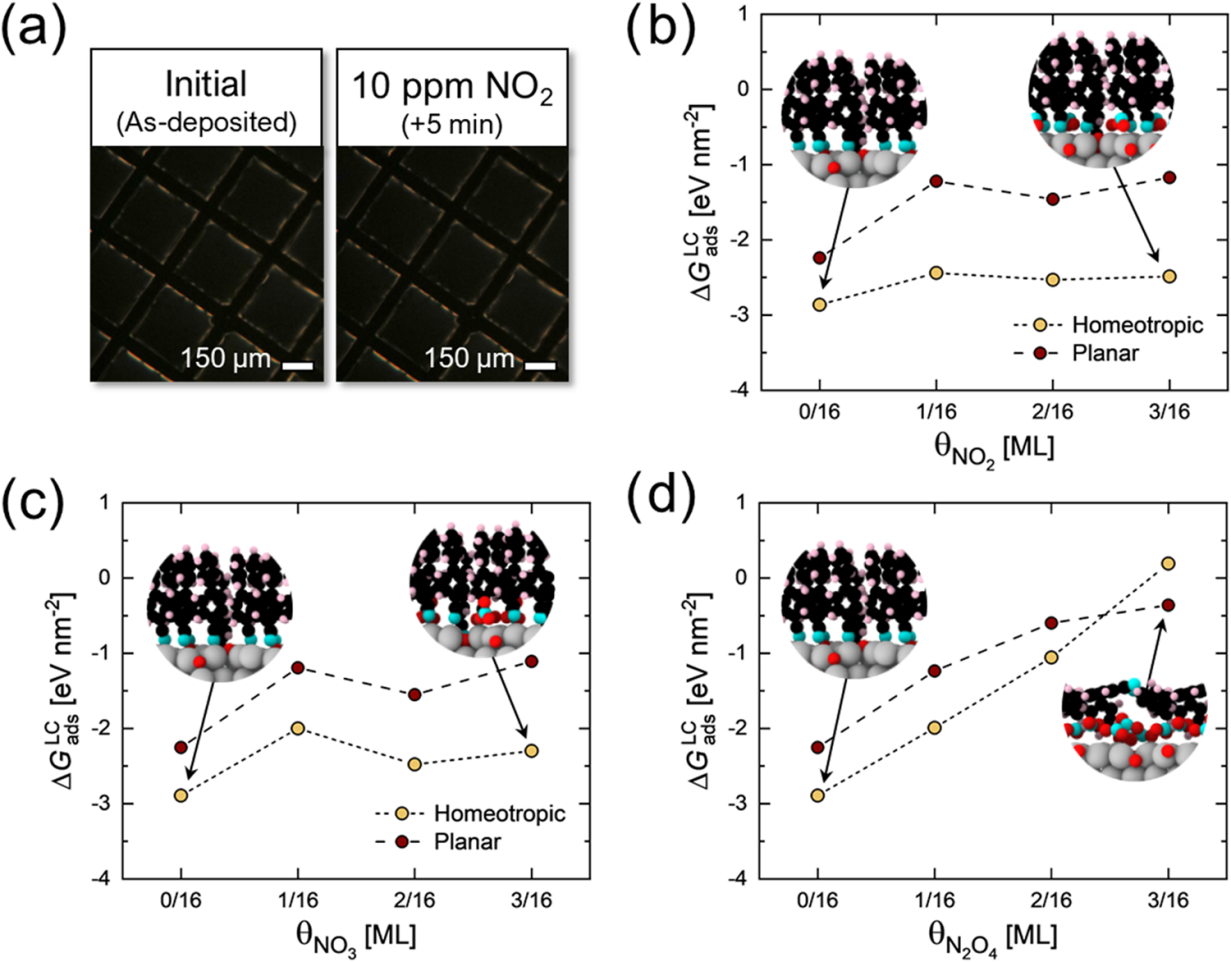
(a) Optical micrographs of 5CB supported on 30 ML Ag films electrochemically deposited on Au, which were then exposed to 10 ppm of NO_2_ for 5 min. White scale bars correspond to 150 μm. (b–d) Calculated adsorption free energy per unit area (Δ*G*_ads_^LC^) for PhPhCN in perpendicular (5/16 ML PhPhCN) and parallel (2/16 ML PhPhCN) orientations on *p*(4 × 4)-O/Ag(111) as a function of NO_2_, NO_3_, and N_2_O_4_ coverage (θ_NO_*x*__) at 298.15 K. The following atomic color scheme was adopted: H (pink), C (black), O (red), N (cyan), and Ag (silver).

Exposure to 10 ppm of NO_2_ produced no discernible change in the orientational anchoring of the 5CB, with the 5CB films remaining optically dark under white light, consistent with persistent perpendicular alignment (Figure 5a). The same result was obtained for 5CB films exposed to NO_2_ on 20-nm-thick electron-beam evaporated Ag films (Figure S7). To investigate the apparent lack of response of 5CB to NO_2_ exposure, we performed DFT calculations to examine the influence of NO_2_, NO_3_, and N_2_O_4_ on the adsorption energetics of PhPhCN on *p*(4 × 4)-O/Ag(111). As established previously, these species exist in non-negligible equilibrium coverages on AgO surfaces exposed to NO_2_ under ambient conditions.^35^ At coverages up to 3/16 ML NO_2_ or NO_3_, we observe that adsorption of either NO_2_ or NO_3_ along with PhPhCN results in largely invariant adsorption energies for PhPhCN in either parallel or perpendicular orientations, with the perpendicular orientation of PhPhCN being favored regardless of the NO_*x*_ coverage (Figure 5b,c). Perpendicular anchoring of PhPhCN is also predicted for AgO with coverages of 1/16 and 2/16 ML N_2_O_4_ (Figure 5d). However, at higher N_2_O_4_ coverages of 3/16 ML, the preferred orientation of PhPhCN shifts to a parallel binding mode (Δ*G*_ads_^LC^ = −0.35 eV nm^–2^), with the perpendicular binding mode heavily destabilized (Δ*G*_ads_^LC^ = +0.22 eV nm^–2^).

To assess which NO_*x*_ coverages on the *p*(4 × 4)-O/Ag(111) surface are thermodynamically accessible, we constructed an *ab initio* phase diagram for NO_2_, NO_3_, and N_2_O_4_ adsorption on *p*(4 × 4)-O/Ag(111) predecorated by 5/16 ML PhPhCN (perpendicular orientation; see Section 2 for PhPhCN coverage selection), reflecting our experimental exposure conditions (Figure 6a). At 298.15 K and 10 ppm of NO_2_, and in the presence of perpendicularly aligned PhPhCN, direct NO_2_ adsorption (green trace, Figure 6a) emerges as the most thermodynamically favorable process on the specific AgO surface, yielding a preferred surface coverage of 3/16 ML NO_2_. In contrast, both the formation of the most favorable coverage of NO_3_, via oxidation of NO_2_ by lattice oxygen (dark blue trace, 2/16 ML NO_3_; Figure 6a), or NO_2_ dimerization to N_2_O_4_ (light blue trace, 2/16 ML N_2_O_4_; Figure 6a), yield more positive surface free energies. Under the experimental conditions of Figure 5a, our results therefore suggest that NO_2_ is the principal surface-bound species in the presence of 5CB LCs.

**Figure 6:**
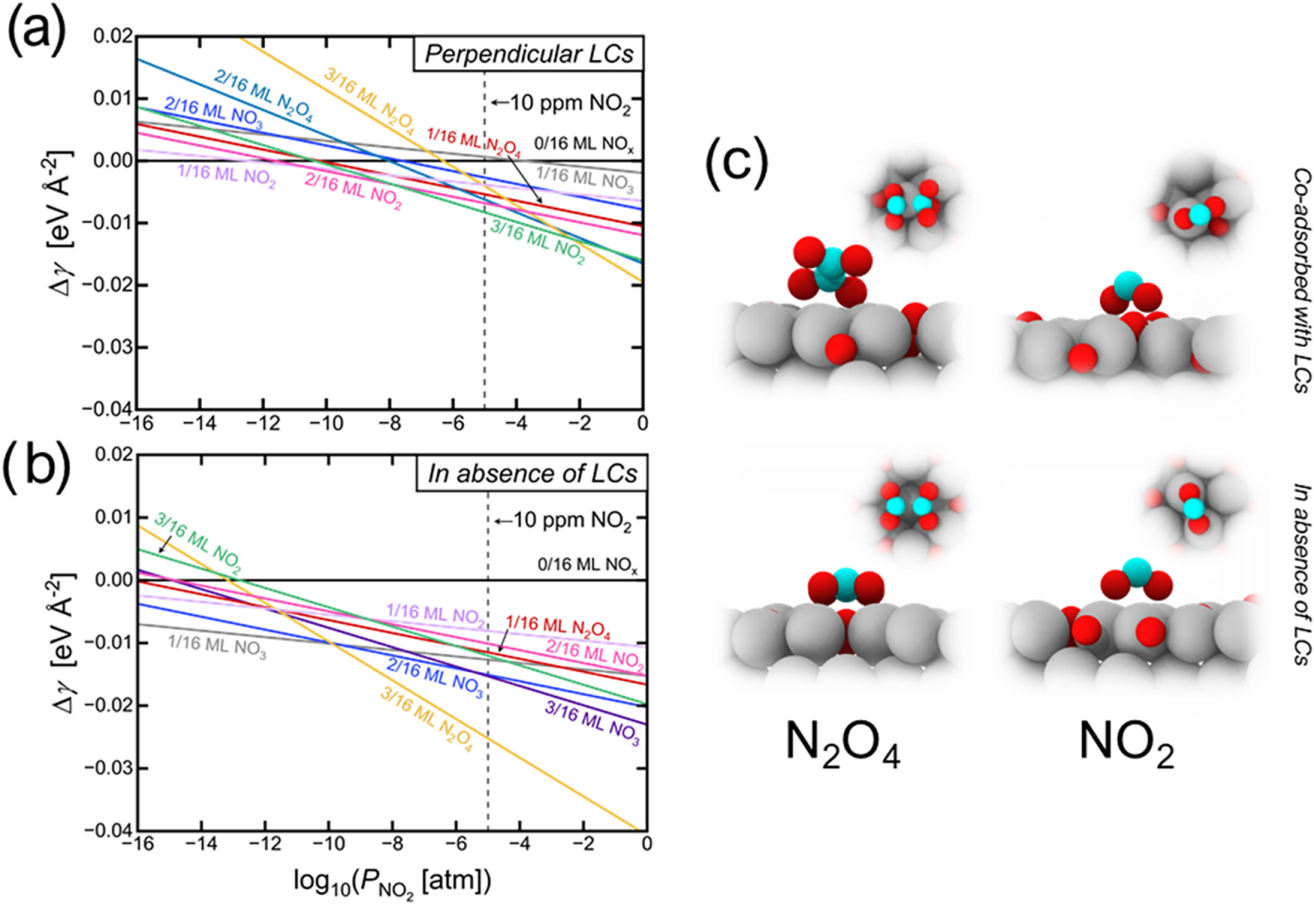
Calculated surface free energy (Δγ) of *p*(4 × 4)-O/Ag(111) (a) decorated with 5/16 ML (homeotropic) PhPhCN and (b) in the absence of PhPhCN as a function of NO_2_, NO_3_, and N_2_O_4_ coverage (1/16 to 3/16 ML) at 298.15 K. The horizontal black line denotes the *p*(4 × 4)-O/Ag(111) surface in the absence of any adsorbed NO_*x*_. Coverages of NO_2_, NO_3_, and N_2_O_4_ thermodynamically inaccessible under any NO_2_ partial pressure are omitted for clarity. The vertical dashed line marks the experimental exposure conditions (10 ppm of NO_2_ at ambient pressure). For N_2_O_4_, we reference the experimental gas-phase chemical potential of NO_2_, assuming dimerization to form N_2_O_4_.^35^ For NO_3_, we reference the experimental gas-phase chemical potential of NO_2_ ^48^ and the chemical potential of the least strongly bound oxygen atom in the LC-decorated *p*(4 × 4)-O/Ag(111) surface layer, assuming NO_3_ formation through reaction of NO_2_ with surface oxygen. (c) Adsorption modes of N_2_O_4_ and NO_2_ on *p*(4 × 4)-O/Ag(111) terrace sites, either coadsorbed with 5/16 ML PhPhCN in a perpendicular orientation or in the absence of PhPhCN. The larger panels display cross-sectional slab views, with corresponding top views shown as insets. For clarity, only a single NO_*x*_ species is depicted in each structure, with other adsorbates omitted. For NO_2_, the global minimum adsorption configuration corresponds to binding within an Ag surface vacancy; however, only the preferred terrace adsorption geometry on Ag sites on the *p*(4 × 4) surface is shown, as surface vacancies constitute a minor fraction of available binding sites. The following atomic color scheme was adopted in the inset: H (pink), O (red), N (cyan), and Ag (silver).

Although our prior XPS measurements and DFT calculations indicate that N_2_O_4_ can form in high coverages on AgO surfaces in the absence of LCs (phase diagram without LC surrogates shown in Figure 6b),^35^ our calculations here indicate that N_2_O_4_ only approaches a surface coverage of 2/16 ML (light blue trace, Figure 6a) in the presence of LCs. This lies below the critical 3/16 ML threshold identified in Figure 5d for inducing a planar-to-perpendicular transition in PhPhCN orientation. Furthermore, while our surface speciation model assumes that oxygen for NO_3_ formation originates from the AgO lattice, which is reasonable given the nitrogen-balanced NO_2_ exposure stream, our conclusions are not sensitive to this choice of reference. When molecular O_2_ is instead taken as the oxygen source,^48^ NO_3_ emerges as the dominant surface species in the presence of coadsorbed PhPhCN (Figure S10) and continues to promote perpendicular alignment (Figure 5c). These theoretical results offer two possible explanations for the lack of response of 5CB (perpendicular ordering) when the oxidized Ag surfaces supporting 5CB are exposed to 10 ppm of NO_2_.

We propose that adsorption of 5CB on *p*(4 × 4)-O/Ag(111) shifts the thermodynamically preferred NO_*x*_ species from N_2_O_4_ to NO_2_ by perturbing the adsorption geometry of the former (Figure 6c). Whereas NO_2_ maintains a bidentate configuration on terrace sites both in the absence and presence of perpendicularly aligned PhPhCN, N_2_O_4_ undergoes a pronounced structural rearrangement in the presence of coadsorbed LCs, transitioning from a flat, multidentate geometry, where all four oxygen atoms interact with surface Ag atoms, to a tilted configuration with two oxygen atoms oriented toward the vacuum. This adsorbate-selective destabilization is consistent with the relative adsorption energetics of the three species. Previous calculations report binding energies of −1.21 eV for NO_2_ and −0.54 eV for N_2_O_4_ on *p*(4 × 4)-O/Ag(111) (GGA-PBE-D3; 1/16 ML NO_*x*_ coverage).^35^ In comparison, we calculate that perpendicularly oriented PhPhCN binds with an average adsorption free energy of −0.66 eV/molecule at 5/16 ML PhPhCN coverage. These energetics are consistent with competitive displacement of N_2_O_4_, but not NO_2_, from Ag surface sites by PhPhCN, consistent with the reduction in Ag–O coordination observed for N_2_O_4_ upon coadsorption of PhPhCN (Figure 6c).

### 3.4 Response of 5CB to NO_2_ over Copper Surfaces

Finally, we present results for the response of 5CB to NO_2_ supported on as-deposited Cu oxide surfaces, which have been shown capable of undergoing oxidation through NO_2_* decomposition into NO*, N*, and O*.^18^ Motivated by this prior work, we performed XPS measurements (Figure 7a,b) to characterize 30 ML Cu/Au films before and after exposure to 10 ppm of NO_2_. From the Cu 2p spectrum, two peaks corresponding to Cu^+^ in Cu_2_O at 931.9 and 951.7 eV can be discerned prior to NO_2_ exposure (I in Figure 7a).^18,71,72^ Additionally, weak intensity peaks at 934.3 and 954.1 eV indicate that, while the Cu film is dominated by Cu_2_O domains, the surface is not morphologically homogeneous, with Cu atoms in regions of the surface existing in higher, 2+ oxidation states.^18,71,72^ Exposure of the Cu film to 10 ppm of NO_2_ (II in Figure 7a) resulted in a growth in intensity of peaks ascribed to Cu^2+^ and the appearance of high-intensity satellite peaks at 941.9, 944.0, and 962.6 eV. Signatures of Cu^+^ at 931.8 and 951.6 eV persisted in the post-exposure surface, indicating incomplete oxidation of the Cu film. From the N 1s spectrum following NO_2_ exposure, low-intensity features (see below for additional discussion) consistent with chemisorbed N, NO, and physisorbed NO_2_ were observed at 399.4, 401.8, and 406.5 eV, respectively (Figure 7b).^18^ Signatures for these species have previously been reported at ambient temperature and a similar NO_2_ partial pressure following the oxidation of Cu_2_O­(111) to CuO(111) (10^–4^ Torr).^18^ Together with the XPS spectra presented in Figure 2c,d, which indicated that oxidation is confined to the near-surface region of the Cu film, the XPS spectra in Figure 7a,b suggest that the surface structure of the 30 ML Cu/Au film exists as majority Cu_2_O domains prior to NO_2_ exposure. These Cu_2_O domains then undergo oxidation to form mixed domains of CuO/Cu_2_O following ambient exposure to 10 ppm of NO_2_.

**Figure 7:**
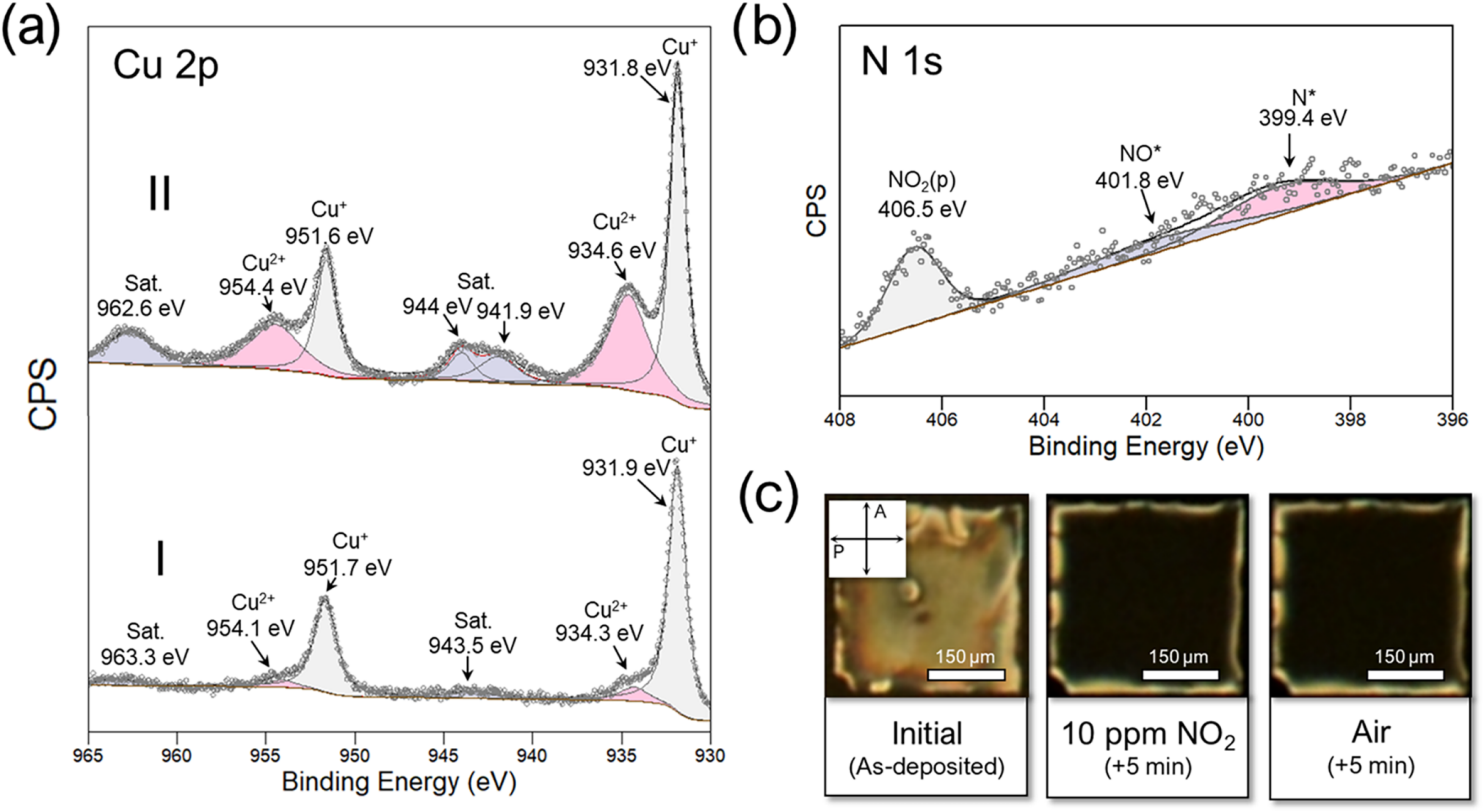
(a) Cu 2p X-ray photoelectron spectra for 30 ML Cu films deposited on 20 nm Au films pre- (I) and post-exposure (II) to 10 ppm of NO_2_. The Cu film shown in (II) was exposed to 10 ppm of NO_2_ for 30 min in a glovebag and transferred into an XPS chamber without subsequent exposure to the atmosphere. Peaks assigned to Cu^+^, Cu^2+^, and satellite peaks are shaded in gray, pink, and purple, respectively. (b) N 1s spectrum post-exposure to 10 ppm of NO_2_ for 30 min in a glovebag and transferred into an XPS chamber without subsequent exposure to the atmosphere. Peaks assigned to physisorbed NO_2_ (NO_2_(*p*)), N*, and NO* are shaded in gray, pink, and purple, respectively. (c) Optical micrographs (crossed polars; top view) showing 20-μm-thick films of 5CB supported on 30-ML-thick Cu/Au films, which were exposed to 10 ppm of NO_2_ for 5 min and then air for another 5 min.

Having determined the dominant surface structure of the Cu film before and after exposure to NO_2_, we next exposed nematic 5CB films supported on 30 ML of the Cu/Au film to 10 ppm of NO_2_ to investigate the effect of the Cu oxidation state on the orientation of LCs. In the absence of NO_2_ exposure, 5CB adopts a planar orientation on the Cu_2_O-dominated Cu film, as shown through a pink and green birefringent texture generated by white light illumination in Figure 7c. In agreement with our experimental observations, DFT calculations for the adsorption of PhPhCN on Cu_2_O­(111) in Table 1 indicated that a planar binding mode was 0.52 eV nm^–2^ more stable than perpendicular binding.

**Table 1:** DFT-Calculated Adsorption Free Energies Normalized Per Unit Area (Δ*G*_ads_) for Perpendicular and Planar Adsorption of 5CB Surrogate Molecules (PhPhCN) on Cu_2_O­(111) and CuO(111) at 298.15 K^a^

	Δ*G*_ads_^LC^ [eV/nm^2^]		
surface	perpendicular	planar	predicted anchoring
Cu_2_O(111)	–4.35	**–4.87**	planar
CuO(111)	**–2.38**	–2.30	perpendicular

a LC coverages of 6/16 ML and 4/16 ML were sampled for perpendicular and planar LC orientations, respectively.

Upon exposure to 10 ppm of NO_2_, we observed a bright (planar) to dark (perpendicular) optical transition of the 5CB film (Figure 7c), consistent with a perpendicular end state for the LC’s anchoring. The perpendicular orientation of 5CB after exposure to NO_2_ remained unchanged for at least 1 h when subsequently exposed to a stream of air at room temperature. This result, coupled with the XPS photoemission spectra in Figure 7a, indicates that 5CB undergoes an orientational transition following oxidation of the Cu film from Cu^+^ to Cu^2+^. Indeed, DFT calculations show that the computational PhPhCN surrogate for 5CB assumes a planar orientation on Cu_2_O­(111) (pre-NO_2_ exposure; Table 1) and a perpendicular orientation on CuO(111) (post-NO_2_ exposure; Table 1).

We note that the DFT-predicted orientation of 5CB is not expected to be strongly influenced by NO_*x*_ coadsorption. Independent ambient-pressure XPS (AP-XPS) measurements by Karagoz et al. of Cu surfaces exposed to 10^–4^ Torr NO_2_ report only weak signals corresponding to nitrogen-based adsorbates, indicating low steady-state NO_*x*_ coverages under conditions where NO_2_ is present in the headspace.^18^ Although our ex situ XPS measurements in Figure 7a,b are performed under vacuum and may underestimate adsorbate coverages relative to the exposure conditions of Figure 7c, the similarly weak signals observed for nitrogen-based adsorbates are consistent with those AP-XPS results.^18^ Furthermore, our analysis of NO_*x*_ adsorption on Ag surfaces (Figure 6a,b) showed that the presence of the LC surrogate reduces, rather than enhances, the thermodynamically preferred NO_*x*_ surface coverage. While minor contributions from NO_*x*_ coadsorption cannot be excluded, the available experimental and theoretical evidence indicates that NO_*x*_ coverages are unlikely to reach levels sufficient to dominate anchoring energetics. The irreversible orientational transition observed in Figure 7c is therefore most consistently explained by the oxidation of Cu^+^ to Cu^2+^ rather than by persistent NO_*x*_ adsorption.

## 4 Conclusion

In summary, this study establishes a framework for understanding surface reactions of NO_2_ on coinage metal surfaces and the interplay between these reactions and the orientations of LC overlayers supported on the surfaces. Using a combined experimental and theoretical approach, we demonstrate that nematic 5CB adopts planar alignment on Au and 30 ML Cu/Au films but adopts a perpendicular (homeotropic) alignment on 30 ML Ag/Au. Upon exposure to 10 ppm of NO_2_ balanced in N_2_ at ambient pressure and temperature, distinct orientational responses emerge from these initial states.

Over Au, favorable electrostatic (dipolar) interactions between adsorbed NO_2_ and perpendicularly aligned 5CB promote the reorientation of the 5CB film from planar to homeotropic alignment upon exposure to NO_2_. Notably, this response on Au is fully reversible upon treatment with synthetic air, underscoring its promise as a highly sensitive and reusable substrate for NO_*x*_ sensing. Although NO_2_ adsorption at room temperature is generally limited to Au defect sites on polycrystalline Au films, our DFT calculations indicate that coadsorbed 5CB enhances NO_2_ binding on Au(111) terraces to a strength comparable to that of defect sites. In this context, 5CB not only acts as a reporter of surface analyte coverage but also promotes the stabilization of an adsorbate that would otherwise fail to bind to pristine Au(111) through favorable dipole–dipole interactions between the LC and the adsorbate. One can therefore envision that other LCs may similarly stabilize adsorbates on solid surfaces, representing a direction for future investigations. If this behavior proves to be general across other combinations of analytes, LCs, and substrates, it could establish a broader molecular design principle in which the LC and supporting surface are selected to selectively stabilize and detect specific analytes. Such an approach would enable the rational design of responsive interfaces for chemical sensing, adaptive surfaces, and chemically driven microactuators.

Over oxide-reconstructed Ag films, 5CB was unresponsive to NO_2_ exposure, with low apparent NO_*x*_ coverages being insufficient for altering the preferred homeotropic alignment of the LC. Analogous to our results over Au films, DFT calculations suggest that coadsorbed 5CB strongly influences both the coverage and speciation of NO_*x*_ on oxide-reconstructed Ag surfaces. Whereas oxide-reconstructed Ag films in the absence of 5CB favor the formation of high N_2_O_4_ coverages, the presence of 5CB shifts the thermodynamically preferred species toward NO_2_ adsorption.

Over Cu, oxidation of the Cu_2_O film to CuO following the reaction and consumption of NO_2_ prompts reorganization of the 5CB film from planar to homeotropic alignment. Although the optical response of 5CB to NO_2_ is irreversible upon exposure to synthetic air, the potential for reversibility via reducing agents such as H_2_, CO, and hydrazine, which are known to reduce CuO films, remains promising for the development of reusable Cu-based NO_*x*_ sensors.

These conclusions, confirmed by polarization-modulation infrared reflection–absorption spectroscopy (PM-IRRAS), X-ray photoelectron spectroscopy (XPS), cross-polarized optical microscopy, and density functional theory (DFT) calculations, directly link atomic-scale surface chemistry to macroscopic LC reorientation. We suggest that these specific insights, guided by predictive computational models, open avenues for designing LC-based materials that optically transduce interfacial reactions with high sensitivity, including chemical sensing applications pertinent to NO_*x*_ detection.

## Supplementary Material



## Notes

Cleaning of glass substrates; XPS and PM-IRRAS measurements; chemoresponsive LC experiments; and density functional theory calculations. (PDF)

## References

[1] Bao N., Gold J. I., Szilvási T., Yu H., Twieg R. J., Mavrikakis M., Abbott N. L. (2021). Designing Chemically Selective Liquid
Crystalline Materials That Respond to Oxidizing Gases. J. Mater. Chem. C.

[2] Bao N., Szilvási T., Tripathi A., Franklin T., Wolter T. J., Shu H., Twieg R. J., Yang R., Mavrikakis M., Abbott N. L. (2025). Design of Chemoresponsive Liquid Crystals Using Metal-Coordinating
Polymer Surfaces. ACS Appl. Mater. Interfaces.

[3] Szilvási T., Roling L. T., Yu H., Rai P., Choi S., Twieg R. J., Mavrikakis M., Abbott N. L. (2017). Design of Chemoresponsive
Liquid Crystals through Integration of Computational Chemistry and
Experimental Studies. Chem. Mater..

[4] Szilvási T., Yu H., Gold J. I., Bao N., Wolter T. J., Twieg R. J., Abbott N. L., Mavrikakis M. (2021). Coupling the Chemical Reactivity
of Bimetallic Surfaces to the Orientations of Liquid Crystals. Mater. Horiz..

[5] Yu H., Gold J. I., Wolter T. J., Bao N., Smith E., Zhang H. A., Twieg R. J., Mavrikakis M., Abbott N. L. (2024). Actuating Liquid Crystals Rapidly and Reversibly by
Using Chemical Catalysis. Adv. Mater..

[6] Yu H., Szilvási T., Wang K., Gold J. I., Bao N., Twieg R. J., Mavrikakis M., Abbott N. L. (2019). Amplification of
Elementary Surface Reaction Steps on Transition Metal Surfaces Using
Liquid Crystals: Dissociative Adsorption and Dehydrogenation. J. Am. Chem. Soc..

[7] Szilvási T., Bao N., Yu H., Twieg R. J., Mavrikakis M., Abbott N. L. (2018). The Role of Anions
in Adsorbate-Induced Anchoring Transitions
of Liquid Crystals on Surfaces with Discrete Cation Binding Sites. Soft Matter.

[8] Shah R. R., Abbott N. L. (2001). Principles for Measurement of Chemical Exposure Based
on Recognition-Driven Anchoring Transitions in Liquid Crystals. Science.

[9] Smith E., Wolter T. J., Twieg R. J., Abbott N. L., Mavrikakis M. (2025). First-Principles
Study of O_2_ Activation over Pd Adclusters and Embedded
Clusters in Pd–Au Bimetallic Surfaces. J. Phys. Chem. C.

[10] Tehrani M. (2021). Advanced Electrical
Conductors: An Overview and Prospects of Metal Nanocomposite and Nanocarbon
Based Conductors. Phys. Status Solidi A.

[11] Serafin J. G., Liu A. C., Seyedmonir S. R. (1998). Surface Science and the Silver-Catalyzed
Epoxidation of Ethylene: An Industrial Perspective. J. Mol. Catal. A.

[12] Porosoff M. D., Yan B., Chen J. G. (2016). Catalytic
Reduction of CO_2_ by H_2_ for Synthesis of CO,
Methanol and Hydrocarbons: Challenges and Opportunities. Energy Environ. Sci..

[13] Haruta M., Kobayashi T., Sano H., Yamada N. (1987). Novel Gold Catalysts
for the Oxidation of Carbon Monoxide at a Temperature Far Below 0
°C. Chem. Lett..

[14] Restrepo C. E. (2021). Nitrogen
Dioxide, Greenhouse Gas Emissions and Transportation in Urban Areas:
Lessons From the Covid-19 Pandemic. Front. Environ.
Sci..

[15] Wickham D. T., Banse B. A., Koel B. E. (1990). Adsorption
of Nitrogen Dioxide on
Polycrystalline Gold. Catal. Lett..

[16] Wu Z., Ma Y., Zhang Y., Xu L., Chen B., Yuan Q., Huang W. (2012). Adsorption and Surface
Reaction of NO_2_ on a Stepped Au(997)
Surface: Enhanced Reactivity of Low-Coordinated Au Atoms. J. Phys. Chem. C.

[17] Sato S., Senga T., Kawasaki M. (1999). Adsorption
States and Photochemistry
of NO_2_ Adsorbed on Au(111). J. Phys.
Chem. B.

[18] Karagoz B., Blum M. A., Head A. R. (2021). Oxidation of Cu_2_O­(111)
by NO_2_: An Ambient Pressure x-Ray Photoelectron Spectroscopy
Study. J. Phys. D: Appl. Phys..

[19] Polzonetti G., Alnot P., Brundle C. R. (1990). The Adsorption
and Reactions of NO_2_ on the Ag(111) Surface II. Adsorption
at 25 K and Annealing
to 300 K. Surf. Sci..

[20] Brown W. A., Gardner P., King D. A. (1995). The Adsorption
of NO_2_ on
Ag {111} : A Low Temperature RAIRS Study. Surf. Sci..

[21] Polzonetti G., Alnot P., Brundle C. R. (1990). The Adsorption and
Reactions of NO_2_ on the Ag(111) Surface. I. XPS/UPS and
Annealing Studies
between 90 and 300 K. Surf. Sci..

[22] Guo X. C., Madix R. J. (2002). Microscopic
Studies of NO_2_ on Ag(110)-p(2
× 1)-O and Reactivity of Surface Nitrate. Surf. Sci..

[23] Alemozafar A. R., Madix R. J. (2005). The Adsorption of and Reaction of
NO_2_ on
Ag(111)-p(4 × 4)-O and Formation of Surface Nitrate. Surf. Sci..

[24] Chidsey C. E. D., Loiacono D. N., Sleator T., Nakahara S. (1988). STM Study
of the Surface
Morphology of Gold on Mica. Surf. Sci..

[25] Baldauf M., Kolb D. M. (1993). A Hydrogen Adsorption and Absorption Study with Ultrathin
Pd Overlayers on Au(111) and Au­(100. Electrochim.
Acta.

[26] Herrero E., Buller L. J., Abruña H. D. (2001). Underpotential
Deposition at Single
Crystal Surfaces of Au, Pt, Ag and Other Materials. Chem. Rev..

[27] Miller D. S., Carlton R. J., Mushenheim P. C., Abbott N. L. (2013). Introduction to
Optical Methods for Characterizing Liquid Crystals at Interfaces. Langmuir.

[28] Hunter J. T., Abbott N. L. (2013). Dynamics of the
Chemo-Optical Response of Supported
Films of Nematic Liquid Crystals. Sens. Actuators,
B.

[29] Kresse G., Furthmüller J. (1996). Efficiency
of Ab-Initio Total Energy Calculations for
Metals and Semiconductors Using a Plane-Wave Basis Set. Comput. Mater. Sci..

[30] Kresse G., Furthmüller J. (1996). Efficient
Iterative Schemes for Ab Initio Total-Energy
Calculations Using a Plane-Wave Basis Set. Phys.
Rev. B.

[31] Perdew J. P., Burke K., Ernzerhof M. (1996). Generalized
Gradient Approximation
Made Simple. Phys. Rev. Lett..

[32] Kresse G., Joubert D. (1999). From Ultrasoft Pseudopotentials to the Projector Augmented-Wave
Method. Phys. Rev. B.

[33] Blöchl P. E. (1994). Projector
Augmented-Wave Method. Phys. Rev. B.

[34] Grimme S., Antony J., Ehrlich S., Krieg H. (2010). A Consistent and Accurate
Ab Initio Parametrization of Density Functional Dispersion Correction
(DFT-D) for the 94 Elements H-Pu. J. Chem. Phys..

[35] Posada-Borbón A., Wolter T., Yu H., Smith E., Schauer J. J., Van Lehn R. C., Zavala V. M., Abbott N. L., Mavrikakis M. (2025). NO_2_ Adsorption on Oxygen-Modified Ag at Ambient Conditions. J. Am. Chem. Soc..

[36] Schmid M., Reicho A., Stierle A., Costina I., Klikovits J., Kostelnik P., Dubay O., Kresse G., Gustafson J., Lundgren E., Andersen J. N., Dosch H., Varga P. (2006). Structure
of Ag(111)-p(4 × 4)-O: No Silver Oxide. Phys. Rev. Lett..

[37] Klacar S., Martin N. M., Gustafson J., Blomberg S., Liu Z., Axnanda S., Chang R., Lundgren E., Grönbeck H. (2013). Facile NOx
Interconversion over Preoxidized Ag(111). Surf.
Sci..

[38] Monkhorst H. J., Pack J. D. (1976). Special Points for Brillouin-Zone Integrations. Phys. Rev. B.

[39] Stukowski A. (2010). Visualization
and Analysis of Atomistic Simulation Data with OVITO-the Open Visualization
Tool. Model. Simul. Mater. Sci. Eng..

[40] Momma K., Izumi F. (2011). VESTA 3 for Three-Dimensional Visualization of Crystal, Volumetric
and Morphology Data. J. Appl. Crystallogr..

[41] Gattinoni C., Michaelides A. (2015). Atomistic Details of Oxide Surfaces
and Surface Oxidation:
The Example of Copper and Its Oxides. Surf.
Sci. Rep..

[42] Scanlon D. O., Morgan B. J., Watson G. W. (2009). Modeling the Polaronic
Nature of
p -Type Defects in Cu_2_O: The Failure of GGA and GGA+*U*. J. Chem. Phys..

[43] Mishra A. K., Roldan A., De Leeuw N. H. (2016). CuO Surfaces
and CO_2_ Activation:
A Dispersion-Corrected DFT+U Study. J. Phys.
Chem. C.

[44] Ekuma C. E., Anisimov V. I., Moreno J., Jarrell M. (2014). Electronic
Structure
and Spectra of CuO. Eur. Phys. J. B.

[45] Bendavid L. I., Carter E. A. (2013). CO_2_ Adsorption
on Cu_2_O­(111):
A DFT+U and DFT-D Study. J. Phys. Chem. C.

[46] Yu X., Zhang X., Wang H., Wang Z., Feng G. (2017). High-Coverage
H_2_ Adsorption on the Reconstructed Cu_2_O­(111)
Surface. J. Phys. Chem. C.

[47] Chiter F., Costa D., Maurice V., Marcus P. (2020). DFT-Based Cu(111)||Cu_2_O­(111) Model for Copper
Metal Covered by Ultrathin Copper
Oxide: Structure, Electronic Properties, and Reactivity. J. Phys. Chem. C.

[48] Chase, M. NIST-JANAF Thermochemical Tables, 4th ed.; American Chemical Society: Washington, DC, 1998; Vol. 9.

[49] Klyushin A. Y., Rocha T. C. R., Hävecker M., Knop-Gericke A., Schlögl R. (2014). A near Ambient Pressure XPS Study of Au Oxidation. Phys. Chem. Chem. Phys..

[50] Barr T. L., Seal S. (1995). Nature of the Use of
Adventitious Carbon as a Binding Energy Standard. J. Vac. Sci. Technol. A.

[51] Huang W., Sun G., Cao T. (2017). Surface Chemistry
of Group IB Metals and Related Oxides. Chem.
Soc. Rev..

[52] de
Mongeot F. B., Valbusa U., Rocca M. (1995). Oxygen Adsorption on
Ag(111). Surf. Sci..

[53] Matsumoto T., Bennett R. A., Stone P., Yamada T., Domen K., Bowker M. (2001). Scanning Tunneling
Microscopy Studies of Oxygen Adsorption
on Cu(111). Surf. Sci..

[54] Weaver J. F., Hoflund G. B. (1994). Surface Characterization
Study of the Thermal Decomposition
of AgO. J. Phys. Chem. A.

[55] Hoflund G. B., Hazos Z. F., Salaita G. N. (2000). Surface
Characterization Study of
Ag, AgO, and Ag_2_O Using x-Ray Photoelectron Spectroscopy
and Electron Energy-Loss Spectroscopy. Phys.
Rev. B.

[56] Gaarenstroom S. W., Winograd N. (1977). Initial and Final State Effects in the ESCA Spectra
of Cadmium and Silver Oxides. J. Chem. Phys..

[57] Turano M.
E., Farber R. G., Oskorep E. C. N., Rosenberg R. A., Killelea D. R. (2020). Characterization
of Oxygenaceous Species Formed by
Exposure of Ag(111) to Atomic Oxygen. J. Phys.
Chem. C.

[58] Bukhtiyarov V. I., Kaichev V. V., Prosvirin I. P. (1999). Oxygen
Adsorption on Ag(111): X-Ray
Photoelectron Spectroscopy (XPS), Angular Dependent x-Ray Photoelectron
Spectroscopy (ADXPS) and Temperature-Programmed Desorption (TPD) Studies. J. Chem. Phys..

[59] Kwan Y. C. G., Ng G. M., Huan C. H. A. (2015). Identification
of Functional Groups
and Determination of Carboxyl Formation Temperature in Graphene Oxide
Using the XPS O 1s Spectrum. Thin Solid Films.

[60] Raukema A., Butler D. A., Box F. M. A., Kleyn A. W. (1996). Dissociative and
Non-Dissociative Sticking of O_2_ at the Ag(111) Surface. Surf. Sci..

[61] Campbell C. T. (1985). Atomic
and Molecular Oxygen Adsorption on Ag­(111. Surf.
Sci..

[62] Poulston S., Parlett P. M., Stone P., Bowker M. (1996). Surface Oxidation
and
Reduction of CuO and Cu_2_O Studied Using XPS and XAES. Surf. Interface Anal..

[63] Chen B., Ma Y., Ding L., Xu L., Wu Z., Yuan Q., Huang W. (2013). XPS and TPD Study of
NO Interaction with Cu(111): Role of Different
Oxygen Species. Chin. J. Catal..

[64] Gupta V. K., Abbott N. L. (1996). Uniform Anchoring
of Nematic Liquid Crystals on Self-Assembled
Monolayers Formed from Alkanethiols on Obliquely Deposited Films of
Gold. Langmuir.

[65] Sen A., Kupcho K. A., Grinwald B. A., Vantreeck H. J., Acharya B. R. (2013). Liquid Crystal-Based Sensors for
Selective and Quantitative
Detection of Nitrogen Dioxide. Sens. Actuators,
B.

[66] Wang J., Koel B. E. (1998). IRAS Studies of
NO_2_, N_2_O_3_, and N_2_O_4_ Adsorbed on Au(111) Surfaces
and Reactions with Coadsorbed H_2_O. J. Phys. Chem. A.

[67] Bartram M. E., Koel B. E. (1989). The Molecular Adsorption
of NO_2_ and the
Formation of N_2_O_3_ on Au(111). Surf. Sci..

[68] Nayani K., Rai P., Bao N., Yu H., Mavrikakis M., Twieg R. J., Abbott N. L. (2018). Liquid Crystals
with Interfacial
Ordering That Enhances Responsiveness to Chemical Targets. Adv. Mater..

[69] Benziger J. B. (1980). Influence
of Dipole Interactions on Surface Reactions. J. Chem. Soc., Faraday Trans. 1.

[70] Deshlahra P., Conway J., Wolf E. E., Schneider W. F. (2012). Influence
of Dipole-Dipole Interactions on Coverage-Dependent Adsorption: CO
and NO on Pt(111). Langmuir.

[71] Kim K., Choi P. G., Itoh T., Masuda Y. (2021). Effect of Coordinatively
Unsaturated Sites in MOF-Derived Highly Porous CuO for Catalyst-Free
Ppb-Level Gas Sensors. Adv. Mater. Interfaces.

[72] Meng W., Yang H., Zhang W., Li Y., Zheng Y., Zhu T. (2025). Surface Sulfurization of Cubic Cu_2_O to Form Cu_2_S Nanostructure-Decorated Catalysts for Light-Enhanced Electrocatalytic
H_2_ Evolution and Photocatalytic CO_2_ Reduction. Chem. – Eur. J..

