# Supplementary material for: Probing NO2 Reactivity on Coinage Metal Surfaces through Liquid Crystal Orientational Responses
Source: J Phys Chem C Nanomater Interfaces. 2026 Jun 22;130(26):9048–61. doi: 10.1021/acs.jpcc.6c02631 (PMC13339829; doi:10.1021/acs.jpcc.6c02631)
Supplement: Supplementary file 1 [file jp6c02631_si_001.pdf]

Supporting Information:

Probing NO_2 Reactivity on Coinage Metal Surfaces through Liquid Crystal Orientational Responses

Evangelos Smith,[†] Huaizhe Yu,[¶] Hanyu Zhang,[¶] Trenton J. Wolter,[†] Alvaro Posada-Borbón,[†] Robert J. Twieg,[‡] Nicholas. L. Abbott,[*,¶] and Manos Mavrikakis[*,†]

[†] *Department of Chemical and Biological Engineering, University of Wisconsin–Madison, Madison, WI 53706, USA.*
[‡] *Department of Chemistry and Biochemistry, Kent State University, 1175 Risman Drive, Kent, Ohio 44242, USA*
[¶] *Robert Frederick Smith School of Chemical and Biomolecular Engineering, Cornell University, 1 Ho Plaza, Ithaca, New York 14853, USA*

***Corresponding Authors:**

Manos Mavrikakis (emavrikakis@wisc.edu, ORCID 0000-0002-5293-5356)

Nicholas L. Abbott (nabbott@cornell.edu, ORCID 0000-0002-9653-0326)

1. Preparation of Substrates

Cleaning of Glass Substrates. Glass microscope slides were cleaned according to published procedures[1] using an acidic piranha solution [70:30 (% v/v) H_2SO_4 (70%):H_2O_2 (30%)]. Piranha solution is extremely corrosive and potentially explosive and should not be exposed to organic material under any circumstances.[2] Briefly, the glass slides were immersed in a piranha bath at 60-80°C for at least 1 hour and then rinsed in running deionized water for 2-3 mins. The slides were then immersed in basic piranha solution [70:30 (%v/v) KOH (45%):H_2O_2 (30%)] and heated to between 60 and 80°C for at least 1 h. Finally, the slides were rinsed sequentially in deionized water and ethanol and then dried under a stream of nitrogen. The cleaned slides were stored in a vacuum oven at 110°C overnight. All glassware was cleaned before use.

Preparation of Gold Substrates for Infrared Spectroscopy. Substrates used in polarization-modulation infrared reflectance absorbance spectroscopy (PM-IRRAS) measurements were prepared by sequential deposition of 100 Å of titanium and 1000 Å of gold onto silicon wafers. The gold-coated silicon wafers were then cut into 15 mm × 30 mm pieces and cleaned under a gaseous stream of nitrogen.

Preparation of Silver and Copper Surfaces.

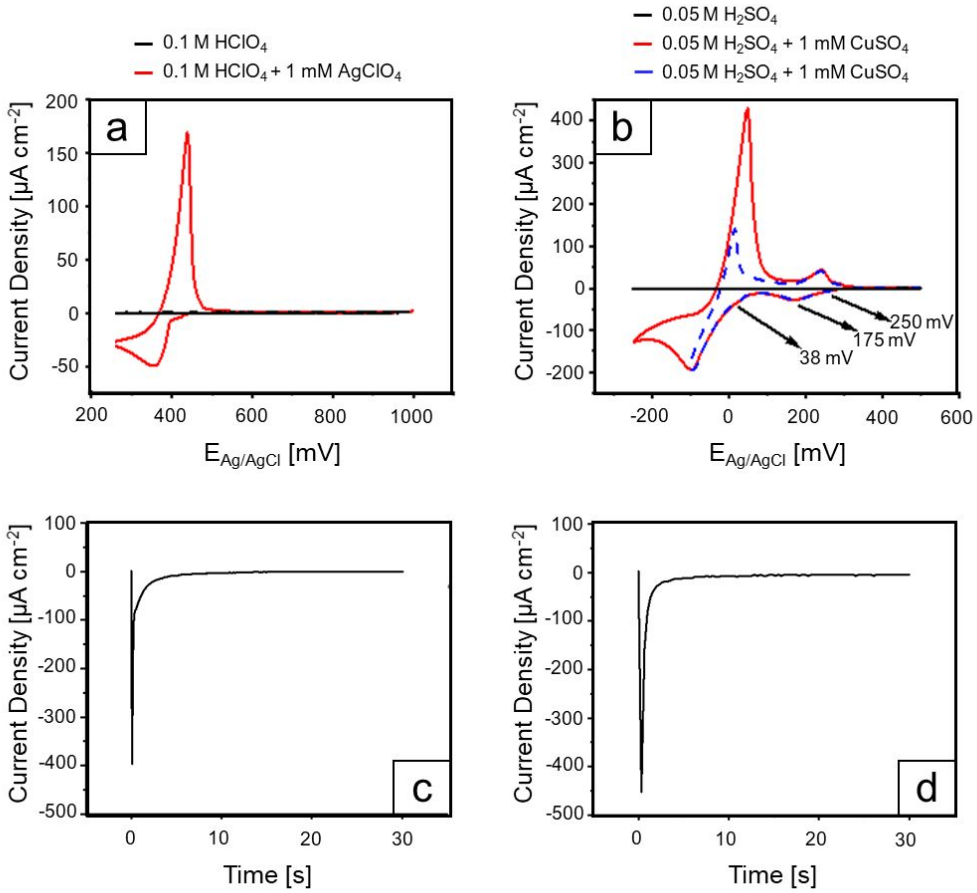

Figure S1. Cyclic voltammograms obtained using Au films in (a) 0.1 M $HClO_4$ + 1 mM $AgClO_4$ (red solid line) and 0.1 M $HClO_4$ (black solid line) and (b) 0.05 M H_2SO_4 + 1 mM $CuSO_4$ with a return at -250 mV (red solid line) and a return at -90 mV (blue dash line) and 0.05 M H_2SO_4 (black solid line). Scan rate: 5 mV/s. Change in current densities over time for (c) Ag deposition at 410 mV and (d) Cu deposition at 40 mV.

2. XPS and PM-IRRAS Measurements

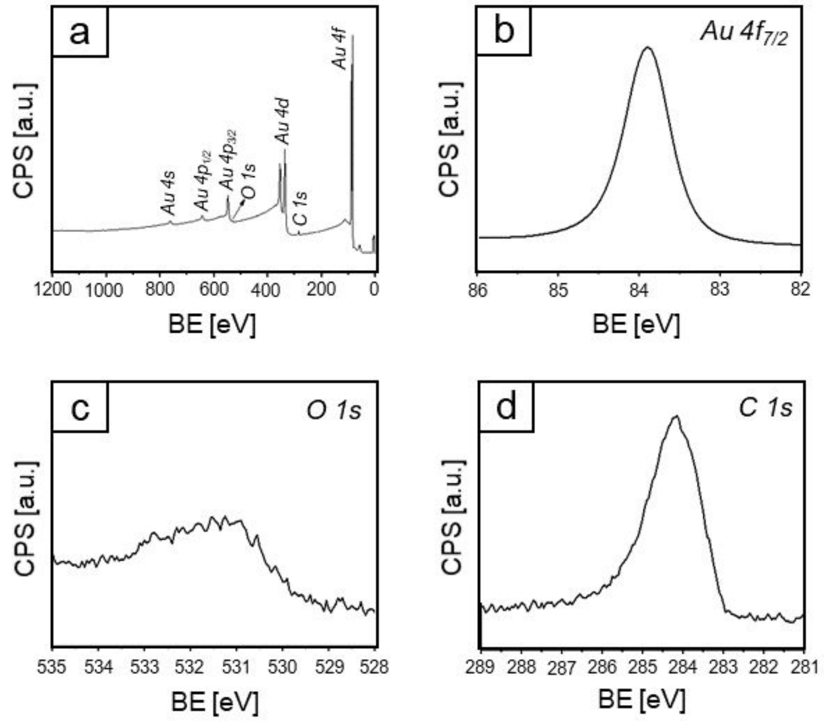

Figure S2. XPS of Au films: (a) survey scan, (b) Au $4f_{72/}$ region, (c) O 1s region and (d) C 1s region.

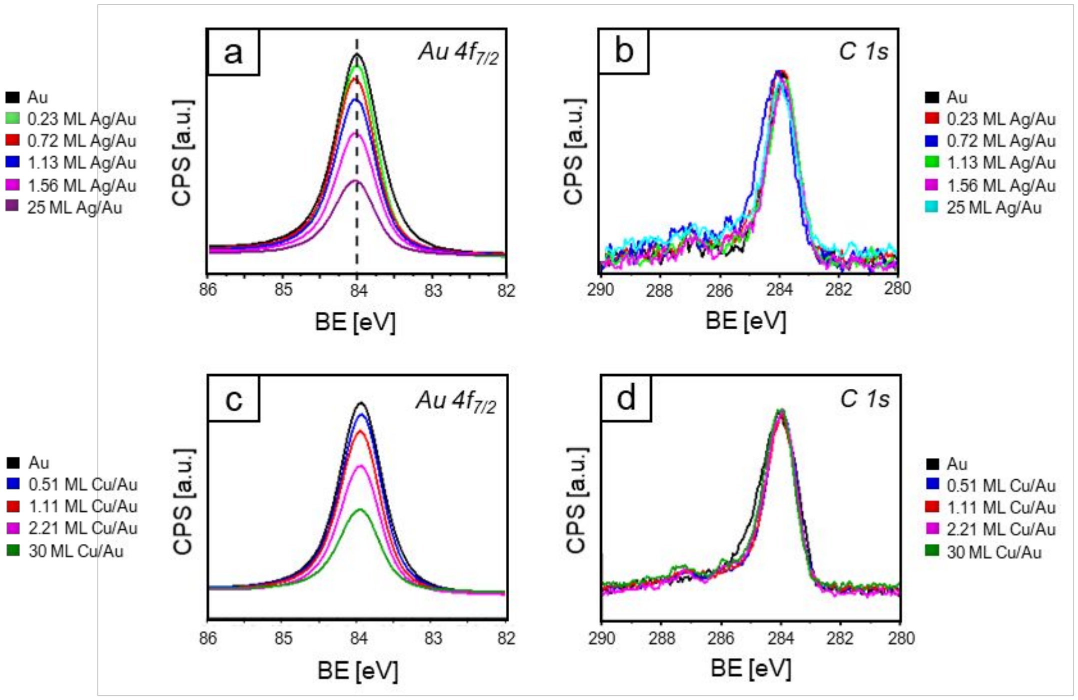

Figure S3. XPS showing the (a) Au $4f_{7/2}$ region and (b) C 1s region for Ag films of varying thicknesses deposited on Au. (c) Au $4f_{7/2}$ region and (d) C 1s region for Cu films of varying thicknesses deposited on Au.

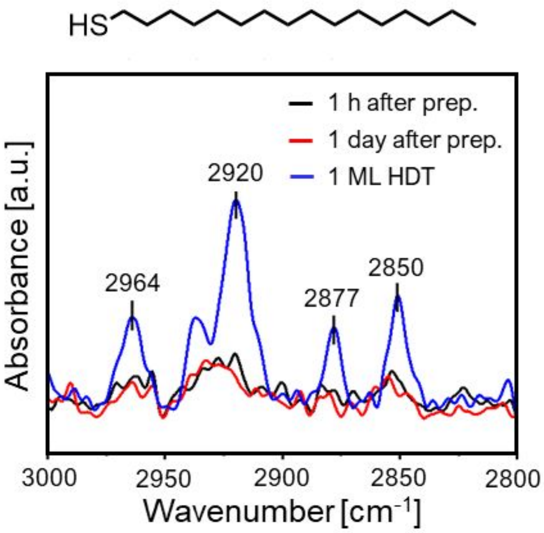

Figure S4. Control experiments showing the PM-IRRAS of 20 nm Au films 1 hour after preparation, 1 day after preparation, and coated with one monolayer of 1-hexadecanethiol (HDT).

3. Chemoresponsive LC Experiments

Experiments on Gold and Copper Films.

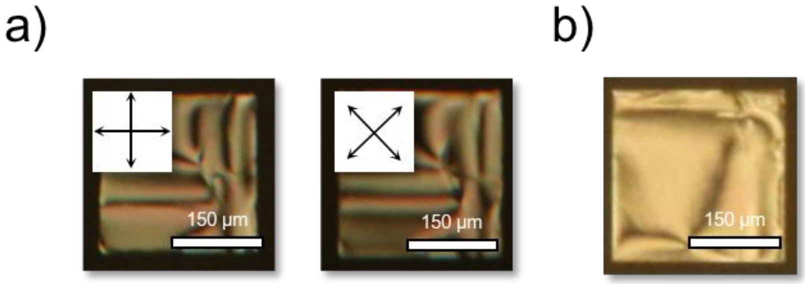

Figure S5. (a) Optical micrographs (crossed polars) of 20-μm-thick films of 5CB supported on Au surfaces at 298 K. The crossed polars were rotated by 45° to obtain the second image. (b) Optical micrograph (crossed polars) of a 20-μm-thick film of 5CB supported on an Au surface that was pre-exposed to 10 ppm NO_2 for 10 min at 298 K before the deposition of 5CB under ambient laboratory atmosphere.

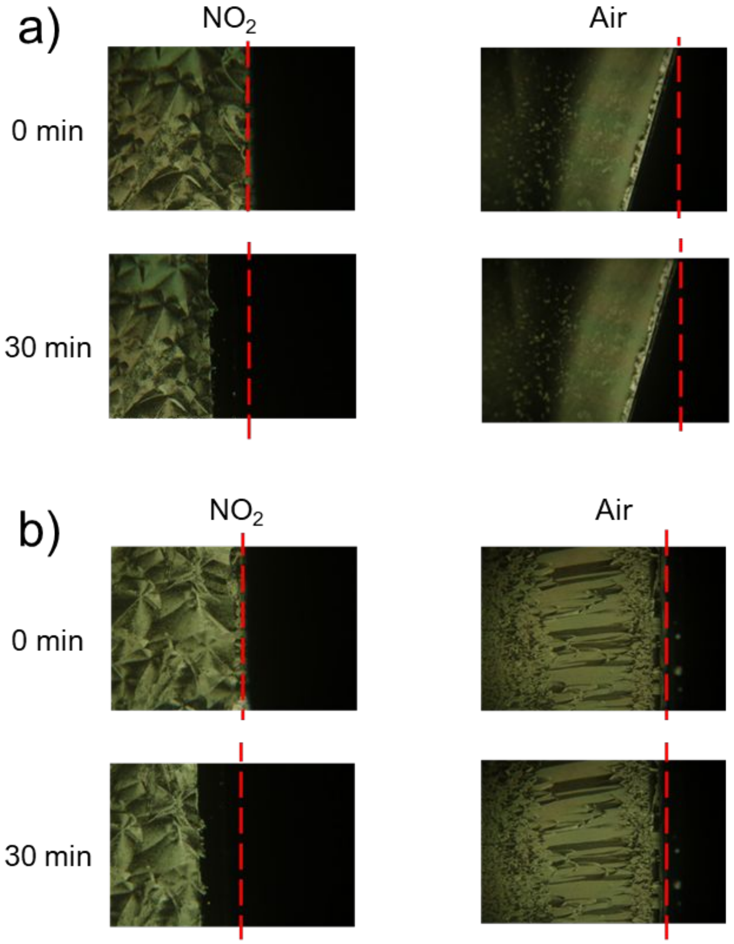

Figure S6. Optical image of sandwich cells made from two (a) Au or (b) Cu substrates and filled with 5CB. The edge of each optical cell (indicated by a dashed red line) was exposed to either 10 ppm NO_2 or a stream of synthetic air (20% O_2/N_2) for 30 min at 298 K.

Experiments on Silver Films. Ag films were also prepared by electron-beam deposition using a CVC SC4500 evaporator at the Cornell Nanoscale Science and Technology facility. 20 nm of Ag was deposited onto a fused silica substrate with a Ti adhesion layer of 2 nm between the Ag and the substrate.

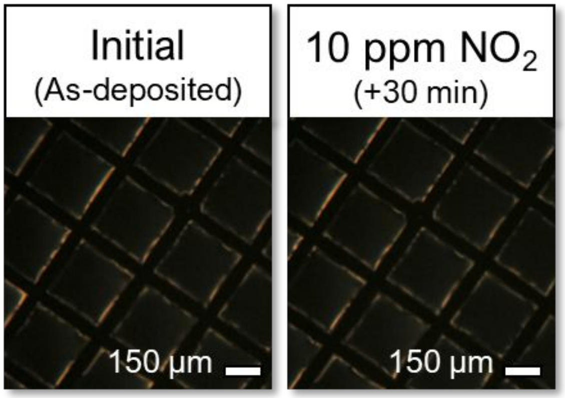

Figure S7. Anchoring experiments on electron-beam-deposited Ag films showing (left) perpendicular anchoring of nematic 5CB, and (right) no change of orientation in the perpendicular 5CB film following exposure to 10 ppm NO_2 for 30 min.

4. Density Functional Theory (DFT) Calculations

PhPhCN as a Computational Surrogate for 5CB. DFT calculations were performed to predict and rationalize the binding orientation of 5CB LC molecules on coinage metal surfaces. 5CB is a mesogenic molecule comprising a biphenyl core with a nitrile headgroup and a pentyl aliphatic tail. For computational efficiency, PhPhCN, a non-mesogenic analogue structurally identical to 5CB but lacking the five-carbon aliphatic tail, was employed as a surrogate to model the anchoring behavior of 5CB at supported metal interfaces. Physically, the aliphatic tail group is expected to interact with metal surfaces weakly via van der Waals interactions,[3] whereas the nitrile headgroup, retained in the surrogate molecule PhPhCN, coordinates directly to the metal surface and, along with the biphenyl core, determines the binding geometry and associated energetics of interest.[4,5] This reduction in molecular size minimizes the computational cost associated with the large unit cells required to accommodate the full 5CB molecular structure and has been shown to qualitatively reproduce experimental anchoring trends of 5CB on supported metal surfaces.[4–6]

PhPhCN Coverage Model. A coverage model for PhPhCN adsorption was established by identifying the number of PhPhCN molecules in a (4×4) surface unit cell which yielded the minimum $\Delta G_{\mathrm{ads}}^{\mathrm{LC}}$ for a given anchoring orientation (Figure S8). For Au(111) and *p*(4×4)-AgO/Ag(111) surfaces, we consider exclusively planar adsorption at a coverage of 2/16 ML of PhPhCN, consistent with our previous studies.[5,6] Due to the considerably larger lattice parameters of Cu_2O and CuO compared to Au and Ag (Table S1), we consider an expanded range of planar/tilted coverages between 2/16 and 4/16 ML PhPhCN and perpendicular coverages of 5/16 and 6/16 ML PhPhCN (Figure S8c,d).

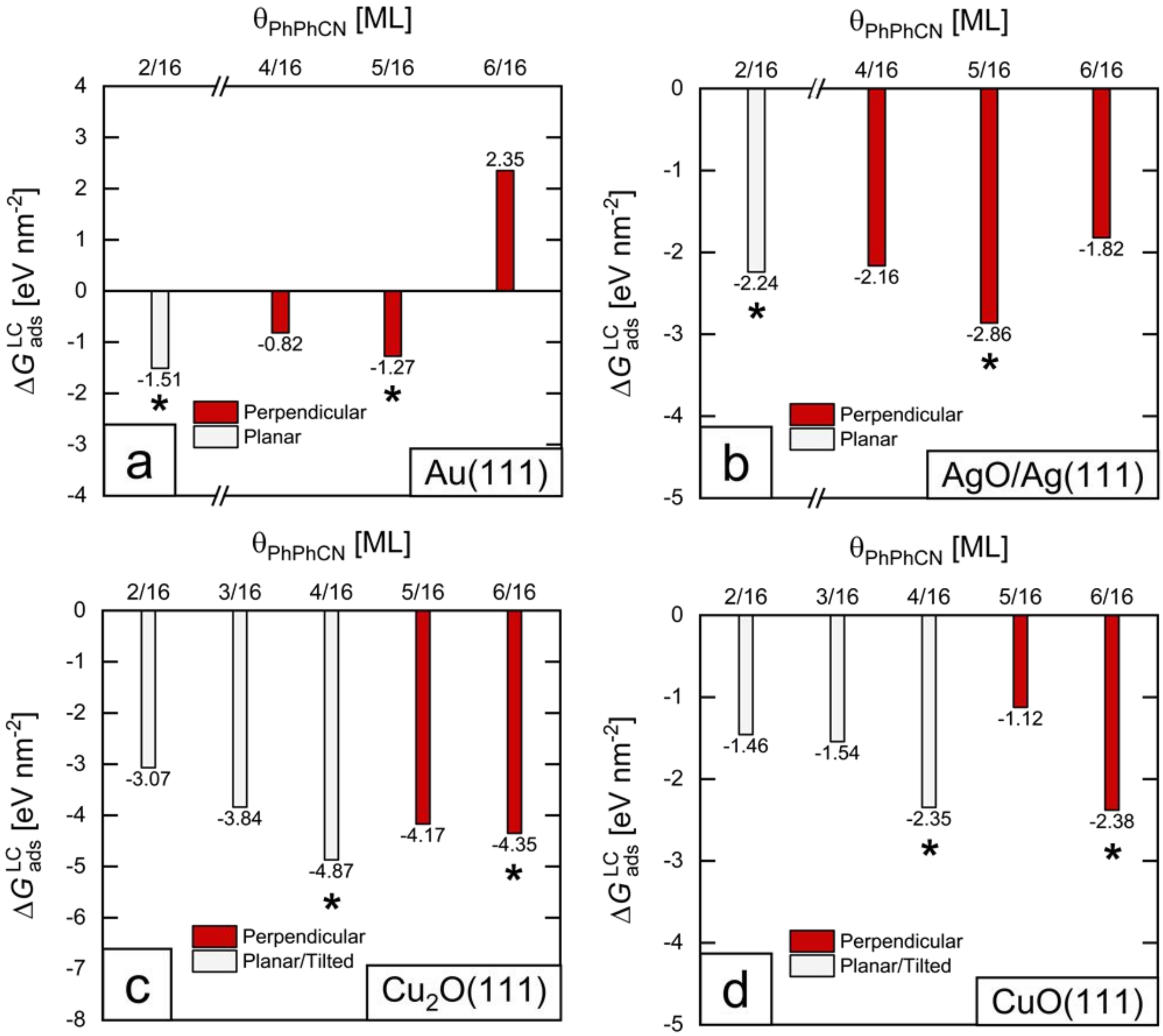

Figure S8. Coverage determination for LC surrogate species. DFT-calculated adsorption free energies normalized per unit area ($\Delta G_{\mathrm{ads}}^{\mathrm{LC}}$) for PhPhCN on pristine (a) Au(111), (b) *p*(4×4)-AgO/Ag(111), (c) Cu_2O(111), and (d) CuO(111). $\Delta G_{\mathrm{ads}}^{\mathrm{LC}}$ of zero denotes PhPhCN molecules in infinite separation from the pristine metal surface. "*" denote the preferred anchoring orientation for perpendicular and planar/tilted alignment.

Table S1. DFT-calculated lattice parameters for Au, Ag, Cu_2O, and CuO.

Metal	*U* Parameter [eV]	Lattice Parameter [Å]	
		DFT	Experimental[a]
Au	-	*a* = *b* = *c* = 4.100	*a* = *b* = *c* = 4.078
Ag	-	*a* = *b* = *c* = 4.072	*a* = *b* = *c* = 4.086
Cu_2O	5.2 (Cu *d*)[b]	*a* = *b* = *c* = 4.241	*a* = *b* = *c* = 4.270
CuO	7.0 (Cu *d*)[c]	*a* = 4.175; *b* = 3.390; *c* = 5.172	*a* = 4.684; *b* = 3.425; *c* = 5.129

[a]Experimental values taken from Ref.[7]
[b]*U* parameter adopted from from Ref.[8]
[c]*U* parameter adopted from from Ref.[9,10]

***Ab Initio* Phase Diagrams.** To assess the thermodynamic stability of NO_x species, we prepared *ab initio* phase diagrams by calculating surface free energies and adsorption free energies of NO_2 on Au(111) and N_2O_4/NO_3 on *p*(4×4)-O/Ag(111). Adsorption/formation free energies ($\Delta G_{\mathrm{ads}}^{\mathrm{NO}_x}$) for NO_2/N_2O_4 on Au(111) or *p*(4×4)-O/Ag(111), as well as NO_3 on *p*(4×4)-O/Ag(111), were calculated using Equations S1 and S2, respectively:

$$\Delta G_{\mathrm{ads}}^{\mathrm{NO}_x} = G_{\mathrm{slab+NO}_x} - G_{\mathrm{slab}} - n_{\mathrm{NO}_2}\mu_{\mathrm{NO}_2} \quad \text{(S1)}$$

$$\Delta G_{\mathrm{ads}}^{\mathrm{NO}_x} = G_{\mathrm{slab+NO}_x} - G_{\mathrm{slab}} - n_{\mathrm{NO}_2}\mu_{\mathrm{NO}_2} - n_{\mathrm{O}}\mu_{\mathrm{O}} \quad \text{(S2)}$$

where $G_{\mathrm{slab+NO}_x}$ is the free energy of NO_2, N_2O_4, or NO_3 adsorbed to the slab (pristine or LC-decorated), G_{slab} is the free energy of the slab, n_{NO_2} and n_{O} are the number of NO_2 and *p*(4×4)-O/Ag(111) lattice oxygen used to form coverages of $NO_2/N_2O_4/NO_3$ per unit cell, and μ_{NO_2} and μ_{O} are the chemical potentials of gas-phase NO_2 and *p*(4×4)-O/Ag(111) lattice oxygen.

The chemical potentials μ_{NO_2} and μ_{O} were calculated using:

$$\mu_{\mathrm{NO}_2} = E_{\mathrm{NO}_2} + \Delta\mathrm{ZPE} + \mu_{\mathrm{NO}_2}^{\mathrm{o}} + k_{\mathrm{B}}T\ln\left(P_{\mathrm{NO}_2}/P^{\circ}\right) \quad \text{(S3)}$$

$$\mu_{\mathrm{O}} = G_{\mathrm{AgO,slab+PhPhCN}} - G_{\mathrm{AgO,slab+PhPhCN+O,vacancy}} \quad \text{(S4)}$$

Here, E_{NO_2} is the DFT-calculated electronic energy of NO_2 in the gas phase, $\Delta\mathrm{ZPE}$ is the zero-point energy correction, *T* and P_{NO_2} are the temperature and NO_2 partial pressure, $\mu_{\mathrm{NO}_2}^{\mathrm{o}}$ includes entropic and enthalpic contributions, and P° is the standard pressure (1 atm). $\mu_{\mathrm{NO}_2}^{\mathrm{o}}$referenced directly to tabulated experimental thermochemical data when constructing phase diagrams,[11] whereas, for internal consistency, it is evaluated within the three-dimensional ideal-gas approximation when calculating NO_2 adsorption energies on Au(111) and Au(874) in the main text. Moreover, $G_{\mathrm{AgO,slab+PhPhCN}}$ is the free energy of *p*(4×4)-O/Ag(111) decorated with 5/16 ML of perpendicularly aligned PhPhCN (initial orientation prior to NO_2 exposure) and $G_{\mathrm{AgO,slab+PhPhCN+O,vacancy}}$ is the free energy of the same PhPhCN-decorated slab with an oxygen vacancy.

Surface free energies (Δγ) were calculated using:

$$\Delta\gamma = \frac{\Delta G_{\mathrm{ads}}^{\mathrm{NO}_x}}{A} \quad \text{(S5)}$$

where *A* is the surface area of the unit cell and $\Delta G_{\mathrm{ads}}^{\mathrm{NO}_x}$ denotes the adsorption energy of NO_x on the LC-decorated metal surface.

NO_2 Adsorption on Au(111). To assess whether Au-supported films of 5CB are predicted to undergo reversible anchoring transitions upon exposure to NO_2, we calculated $\Delta G_{\text{ads}}^{\text{NO}_2}$ for NO_2 adsorption on pristine Au(111) (Figure S9). For NO_2 coverages between 1/16 and 3/16 ML co-adsorbed with 5/16 ML PhPhCN, NO_2 exhibits moderate adsorption free energies (-0.66 to -0.79 eV per NO_2), sufficient to sustain non-zero surface coverages under continued exposure to 10 ppm NO_2 at room temperature. In contrast, at higher NO_2 coverages (>3/16 ML), adsorption weakens substantially, with average adsorption free energies exceeding -0.52 eV per NO_2, indicating diminished surface stability. These trends closely mirror those in Figure 4c, where intermediate NO_2 coverages maximize favorable dipole–dipole interactions between PhPhCN and NO_2 while minimizing unfavorable co-adsorption effects. The moderate-to-weak NO_2 binding energies predicted for PhPhCN-decorated Au(111) are further consistent with experimental observations (Section 3.1 of the main text), in which NO_2 readily desorbs upon sparging with synthetic air, confirming the reversibility of the NO_2-induced anchoring transition.

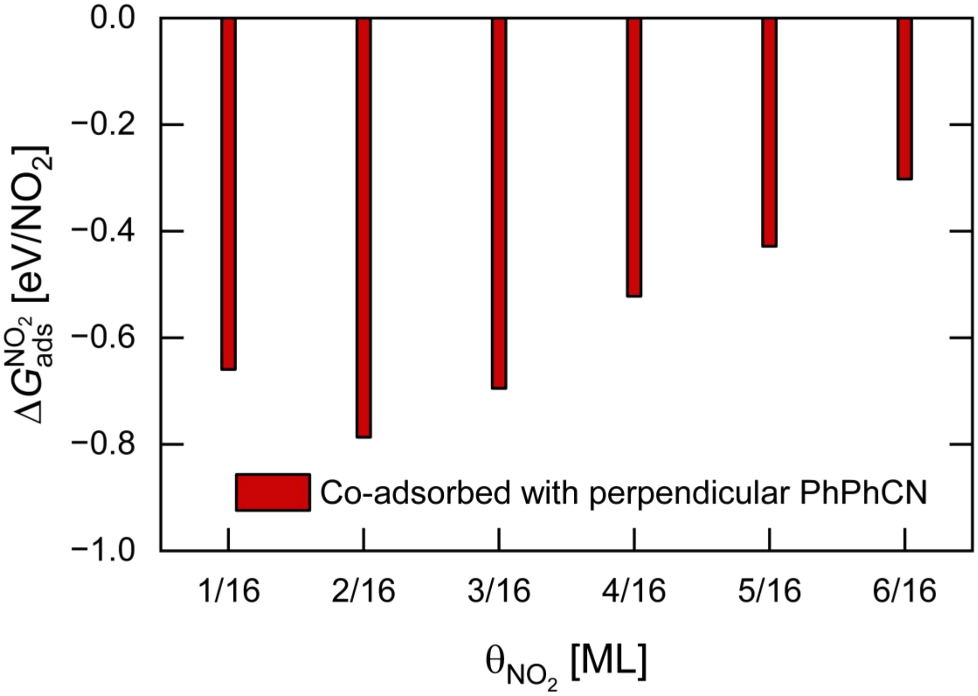

Figure S9. DFT-calculated average adsorption free energies ($\Delta G_{\text{ads}}^{\text{NO}_2}$) for NO_2 on 5/16 ML PhPhCN-decorated Au(111) at 298.15 K and 10 ppm NO_2. $\Delta G_{\text{ads}}^{\text{NO}_2}$ of zero denotes NO_2 in infinite separation from the PhPhCN-decorated Au(111) surface.

NO_x Adsorption/Formation on *p*(4×4)-O/Ag(111).

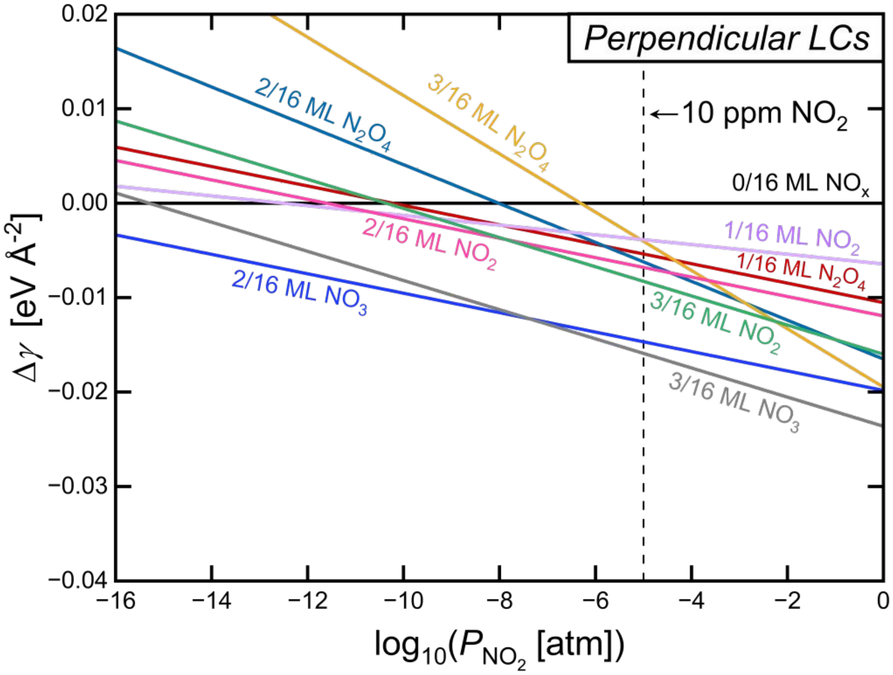

Figure S10. Calculated surface free energy (Δ*γ*) of *p*(4×4)-O/Ag(111) decorated with 5/16 ML (perpendicular) PhPhCN, as a function of NO_2, NO_3, and N_2O_4 coverage (1/16 to 3/16 ML) at 298.15 K. The horizontal black line denotes the LC-decorated *p*(4×4)-O/Ag(111) surface in absence of adsorbed NO_x. Coverages of NO_2, NO_3, and N_2O_4 thermodynamically inaccessible under any NO_2 partial pressure are omitted for clarity. The vertical dashed line marks the experimental exposure conditions (10 ppm NO_2 at ambient pressure). For N_2O_4, we reference the experimental gas-phase chemical potential of NO_2, assuming dimerization to form N_2O_4.[12] For NO_3, we reference the experimental gas-phase chemical potentials of NO_2 and O_2,[11] assuming NO_3 formation through reaction of NO_2 with atomic oxygen.

Adsorption of PhPhCN on Coinage Metal Surfaces.

Table S2. DFT-calculated adsorption free energies normalized per unit cell area ($\Delta G_{\mathrm{ads}}^{\mathrm{LC}}$) for PhPhCN on Au(111) at 298.15 K as a function of NO_2 coverage. For all calculations, the PhPhCN coverage is fixed at 5/16 ML for the perpendicular orientation and 2/16 ML for planar adsorption. The chemical potential of gas-phase PhPhCN is referenced to 1 atm. $\Delta G_{\mathrm{ads}}^{\mathrm{LC}}$ of zero denotes PhPhCN in infinite separation from the pristine or NO_2-decorated Au surface.

$\theta_{\mathrm{NO_2}}$ [ML]	$\Delta G_{\mathrm{ads}}^{\mathrm{LC}}$ [eV nm^{-2}] Perpendicular	Planar	Predicted LC Anchoring
0/16 (Pristine)	-1.27	**-1.51**	Planar
1/16	**-1.45**	-1.44	Perpendicular[a]
2/16	**-2.02**	-1.12	Perpendicular
3/16	**-2.20**	-0.92	Perpendicular
4/16	**-2.07**	-0.84	Perpendicular
5/16	**-1.96**	-0.47	Perpendicular
6/16	**-1.50**	-0.39	Perpendicular

[a]Perpendicular and planar binding orientations of PhPhCN are approximately isoenergetic. The slightly more favorable $\Delta G_{\mathrm{ads}}^{\mathrm{LC}}$ of the perpendicular configuration (by 0.01 eV nm^{-2}) corresponds to a 1.48-fold higher binding probability relative to the planar orientation, assuming a Boltzmann distribution at 298.15 K.

Table S3. DFT-calculated adsorption free energies normalized per unit cell area ($\Delta G_{\mathrm{ads}}^{\mathrm{LC}}$) for PhPhCN on *p*(4×4)-O/Ag(111) at 298.15 K as a function of NO_2, N_2O_4, or NO_3 coverage. For all calculations, the PhPhCN coverage is fixed at 5/16 ML for the perpendicular orientation and 2/16 ML for planar adsorption. The chemical potential of gas-phase PhPhCN is referenced to 1 atm. $\Delta G_{\mathrm{ads}}^{\mathrm{LC}}$ of zero denotes PhPhCN in infinite separation from the pristine or NO_x-decorated Ag surface.

Species	θ_{NO_x} [ML]	$\Delta G_{\mathrm{ads}}^{\mathrm{LC}}$ [eV nm^{-2}] Perpendicular	Planar	Predicted LC Anchoring
Pristine	0/16	**-2.86**	-2.24	Perpendicular
NO_2	1/16	**-2.44**	-1.22	Perpendicular
	2/16	**-2.53**	-1.46	Perpendicular
	3/16	**-2.49**	-1.17	Perpendicular
N_2O_4	1/16	**-1.96**	-1.22	Perpendicular
	2/16	**-1.03**	-0.59	Perpendicular
	3/16	+0.22	**-0.35**	Planar
NO_3	1/16	**-1.97**	-1.18	Perpendicular
	2/16	**-2.45**	-1.54	Perpendicular
	3/16	**-2.27**	-1.10	Perpendicular

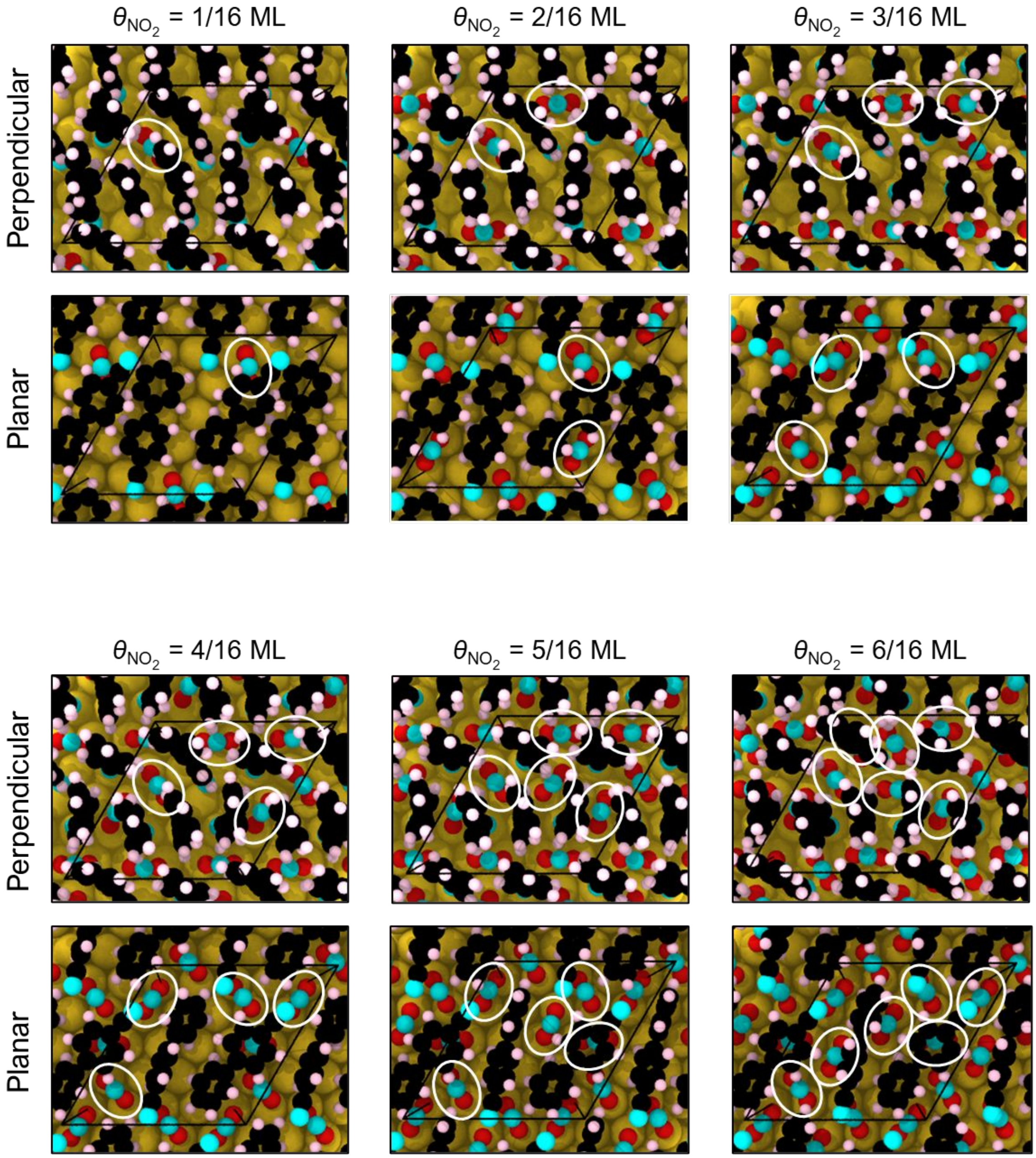

Figure S11. Top views of minimum energy binding modes for PhPhCN on NO_2-decorated Au(111) in a (4×4) unit cell. The following atomic color scheme was adopted: H (pink), C (black), O (red), N (cyan), and Au (gold). The surface unit cell is denoted by black lines, and the coverage (θ_{NO_2}) of NO_2 is indicated above each panel. The positions of unique NO_2 molecules within the unit cell are marked by white circles. The coverage of PhPhCN in perpendicular and planar adsorption modes is 5/16 and 2/16 ML, respectively.

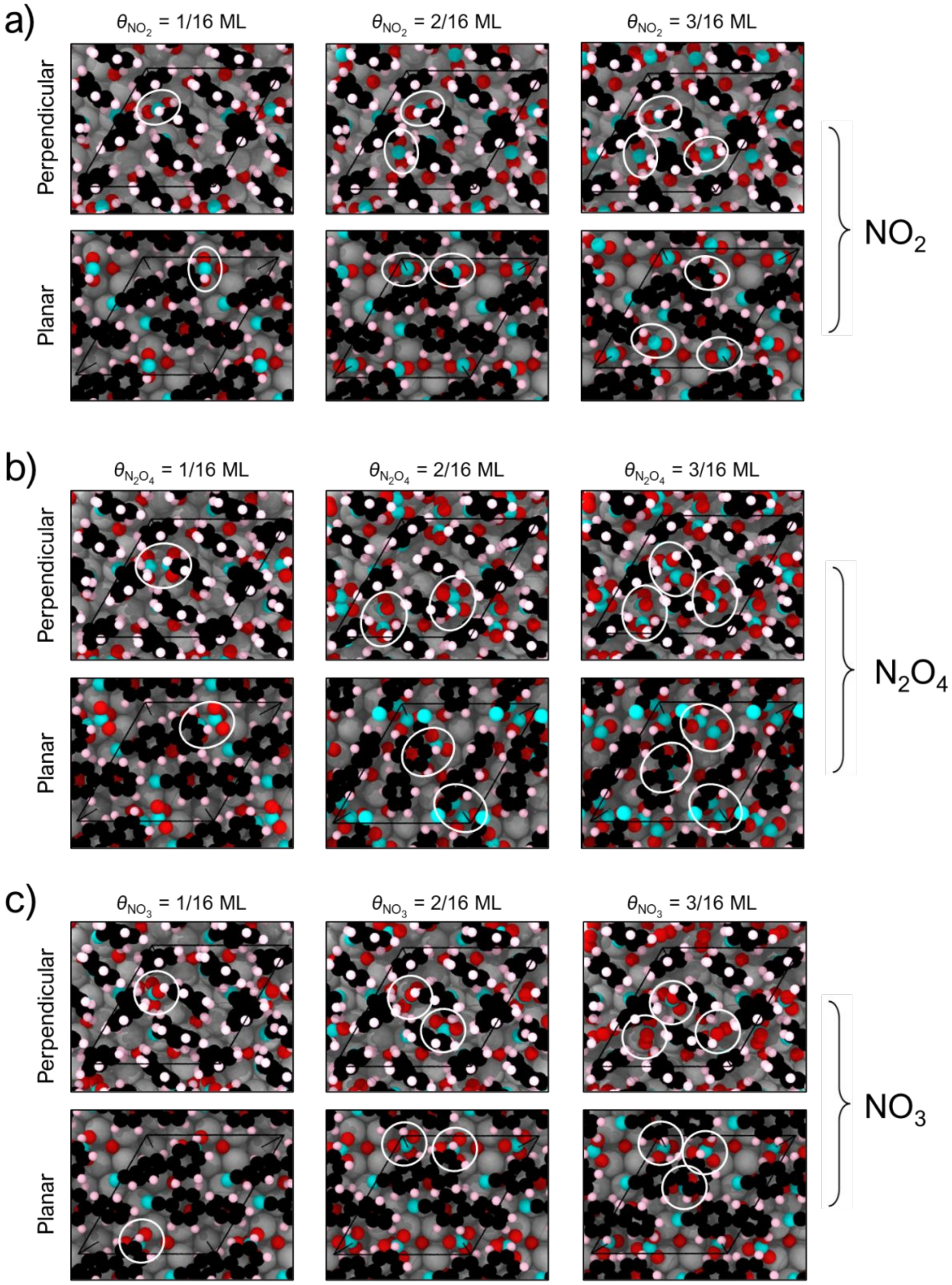

Figure S12. Top views of minimum energy adsorption modes for PhPhCN on (a) NO_2-, (b) N_2O_4- and (c) NO_3-decorated *p*(4×4)-O/Ag(111). The following atomic color scheme was adopted: H (pink), C (black), O (red), N (cyan), and Ag (silver). The surface unit cell is denoted by black lines, and the coverage (θ_{NO_x}) of N_2O_4 or NO_3 is indicated above each panel. The positions of unique NO_x molecules within the unit cell are marked by white circles. The coverage of PhPhCN in perpendicular and planar adsorption modes is 5/16 and 2/16 ML, respectively.

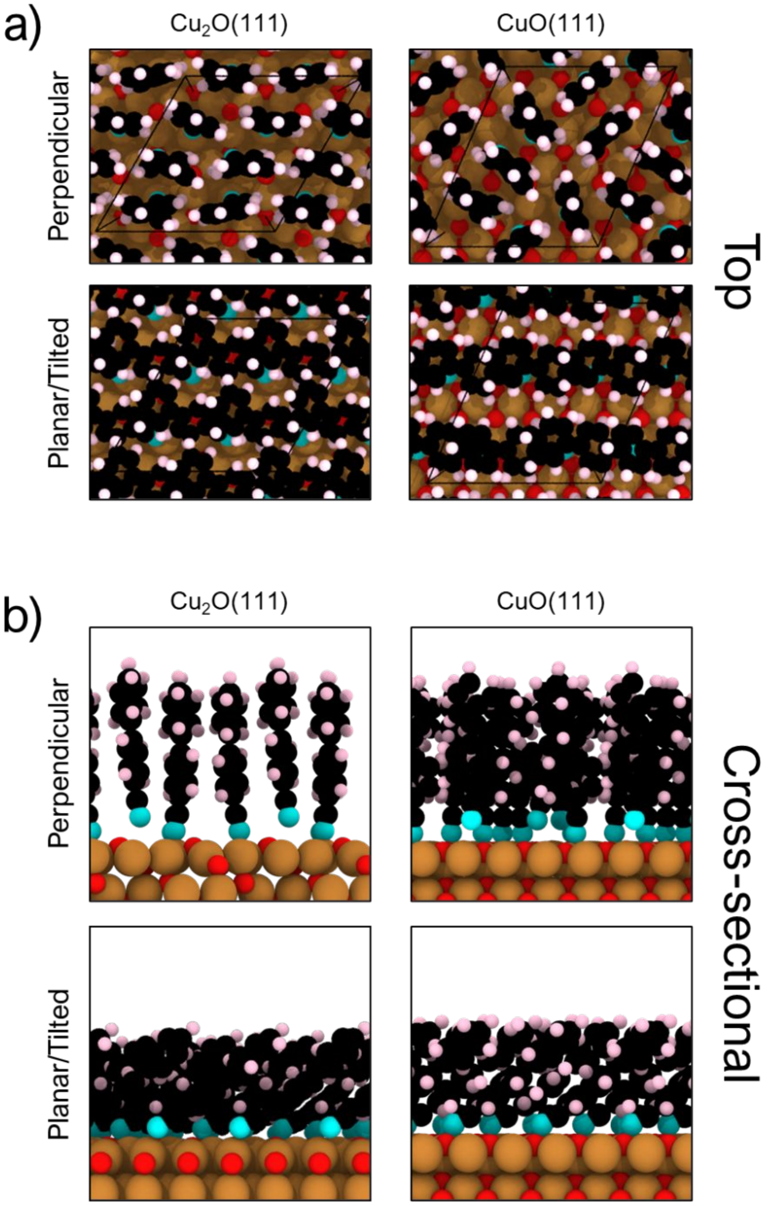

Figure S13. (a) Top and (b) cross-sectional views of minimum energy adsorption modes for PhPhCN on $Cu_2O(111)$ and CuO(111). The following atomic color scheme was adopted: H (pink), C (black), O (red), N (cyan), and Cu (bronze). The surface unit cell is denoted by black lines. The coverage of PhPhCN in perpendicular and planar/tilted adsorption modes is 6/16 and 4/16 ML, respectively.

Calculation of Electrostatic Interactions. Pairwise electrostatic interactions were evaluated following the protocol outlined in Scheme S1. First, fully relaxed unit cells comprised of PhPhCN and NO_2 on Au(111) were generated. Next, each adsorbate was isolated by removing all other adsorbed species from the slab, and its surface-normal dipole moment was computed relative to the adsorbate-slab center of mass. First nearest-neighbor (INN) electrostatic interaction energies ($(\Delta E^{\mathrm{INN}}_{\mathrm{int,A-B}})_i$) were then calculated for each NO_2-PhPhCN and NO_2-NO_2 pair using the isolated adsorbate dipole moments, as defined in Equation 5 of the main text. The total electrostatic interaction energy was obtained by summing $(\Delta E^{\mathrm{INN}}_{\mathrm{int,A-B}})_i$ across all INN pairs in the unit cell. Although this approach omits co-adsorption effects that may alter individual adsorbate dipole moments, it captures essential electrostatic trends for interpreting coverage-dependent trends in the PhPhCN adsorption energies discussed in Section 3.2.

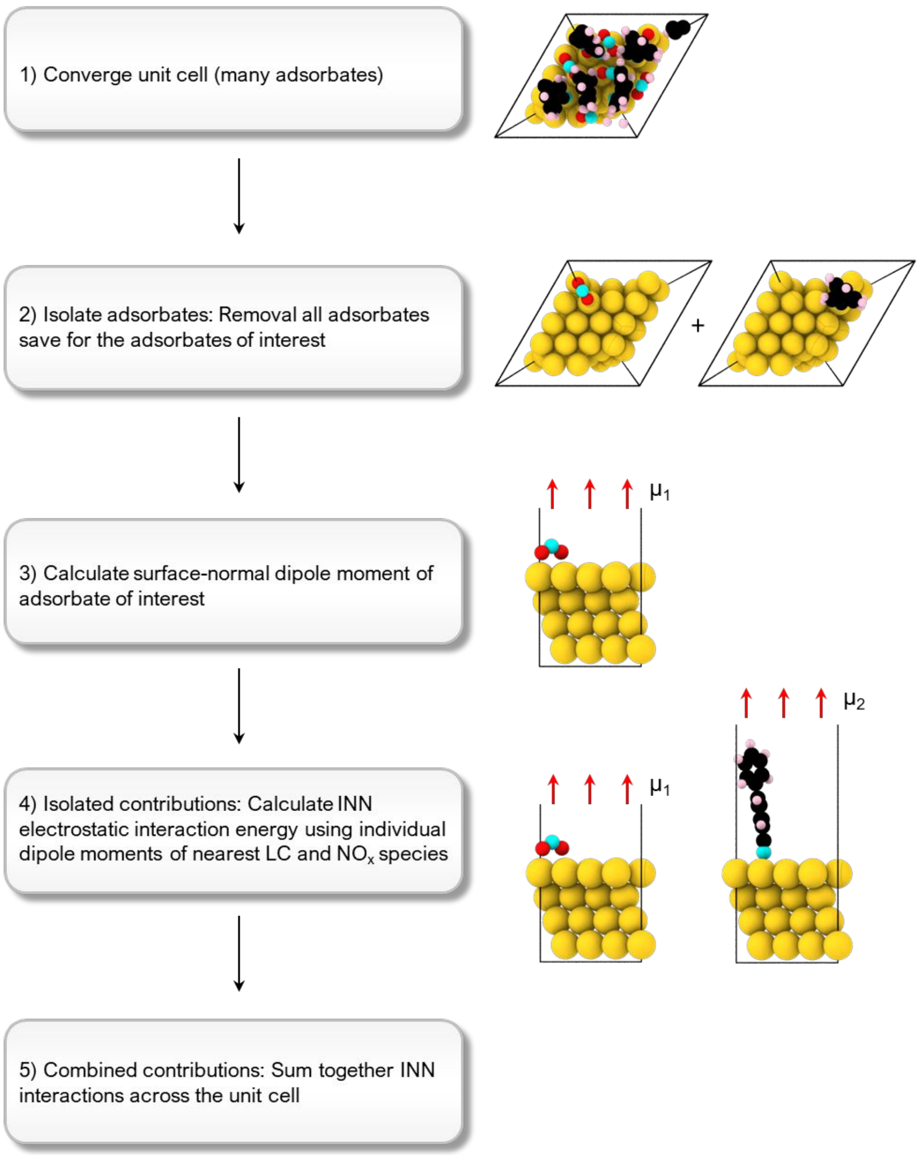

Scheme S1. Protocol established for calculating the overall electrostatic interaction energy between NO_2-PhPhCN and NO_2-NO_2 on Au(111). The following atomic color scheme was adopted: H (pink), C (black), O (red), N (cyan), and Au (gold).

References

(1) Hunter, J. T.; Pal, S. K.; Abbott, N. L. Adsorbate-Induced Ordering Transitions of Nematic Liquid Crystals on Surfaces Decorated with Aluminum Perchlorate Salts. *ACS Appl. Mater. Interfaces* **2010**, *2* (7), 1857–1865. https://doi.org/10.1021/am100165a.

(2) Thomas, S. W.; Vella, S. J.; Dickey, M. D.; Kaufman, G. K.; Whitesides, G. M. Controlling the Kinetics of Contact Electrification with Patterned Surfaces. *J. Am. Chem. Soc.* **2009**, *131* (25). https://doi.org/10.1021/ja902862b.

(3) Xu, L.; Ye, R.; Mavrikakis, M.; Chen, P. Molecular-Scale Insights into Cooperativity Switching of XTAB Adsorption on Gold Nanoparticles. *ACS Cent. Sci.* **2024**, *10* (1), 65–76. https://doi.org/10.1021/acscentsci.3c01075.

(4) Yu, H.; Szilvási, T.; Wang, K.; Gold, J. I.; Bao, N.; Twieg, R. J.; Mavrikakis, M.; Abbott, N. L. Amplification of Elementary Surface Reaction Steps on Transition Metal Surfaces Using Liquid Crystals: Dissociative Adsorption and Dehydrogenation. *J. Am. Chem. Soc.* **2019**, *141* (40), 16003–16013. https://doi.org/10.1021/jacs.9b08057.

(5) Szilvási, T.; Yu, H.; Gold, J. I.; Bao, N.; Wolter, T. J.; Twieg, R. J.; Abbott, N. L.; Mavrikakis, M. Coupling the Chemical Reactivity of Bimetallic Surfaces to the Orientations of Liquid Crystals. *Mater. Horiz.* **2021**, *8* (7), 2050–2056. https://doi.org/10.1039/d1mh00035g.

(6) Yu, H.; Gold, J. I.; Wolter, T. J.; Bao, N.; Smith, E.; Zhang, H. A.; Twieg, R. J.; Mavrikakis, M.; Abbott, N. L. Actuating Liquid Crystals Rapidly and Reversibly by Using Chemical Catalysis. *Adv. Mater.* **2024**, *36* (23), 2309605. https://doi.org/10.1002/adma.202309605.

(7) Haynes, W. M. *CRC Handbook of Chemistry and Physics*, 95th ed.; CRC Press: Boca Raton, 2014. https://doi.org/10.1201/b17118.

(8) Scanlon, D. O.; Morgan, B. J.; Watson, G. W. Modeling the Polaronic Nature of p -Type Defects in Cu_2O: The Failure of GGA and GGA+U. *J. Chem. Phys.* **2009**, *131* (12), 124703. https://doi.org/10.1063/1.3231869.

(9) Mishra, A. K.; Roldan, A.; De Leeuw, N. H. CuO Surfaces and CO_2 Activation: A Dispersion-Corrected DFT+U Study. *J. Phys. Chem. C* **2016**, *120* (4), 2198–2214. https://doi.org/10.1021/acs.jpcc.5b10431.

(10) Ekuma, C. E.; Anisimov, V. I.; Moreno, J.; Jarrell, M. Electronic Structure and Spectra of CuO. *Eur. Phys. J. B* **2014**, *87* (1), 23. https://doi.org/10.1140/epjb/e2013-40949-5.

(11) Chase, M. *NIST-JANAF Thermochemical Tables, 4th Edition*, 4th ed.; American Chemical Society: Washington DC, 1998; Vol. 9.

(12) Posada-Borbón, A.; Wolter, T.; Yu, H.; Smith, E.; Schauer, J. J.; Van Lehn, R. C.; Zavala, V. M.; Abbott, N. L.; Mavrikakis, M. NO_2 Adsorption on Oxygen-Modified Ag at Ambient Conditions. *J. Am. Chem. Soc.* **2025**, *147* (46), 43139–43152. https://doi.org/10.1021/jacs.5c16683.
